# Functional domains of a ribosome arresting peptide are affected by surrounding nonconserved residues

**DOI:** 10.1016/j.jbc.2024.105780

**Published:** 2024-02-22

**Authors:** Heather N.G. Judd, Allyson K. Martínez, Dorota Klepacki, Nora Vázquez-Laslop, Matthew S. Sachs, Luis R. Cruz-Vera

**Affiliations:** 1 Department of Biological Sciences, University of Alabama in Huntsville, Huntsville, Alabama, USA; 2 Department of Biology, Texas A&M University, College Station, Texas, USA; 3 Department of Pharmaceutical Sciences, University of Illinois at Chicago, Chicago, Illinois, USA; 4 Center for Biomolecular Sciences, University of Illinois at Chicago, Chicago, Illinois, USA

**Keywords:** L-Trp, tnaC, peptidyl transferase center, arrested ribosomes, ribosome arresting peptide, ribosomal exit tunnel

## Abstract

Expression of the *Escherichia coli tnaCAB* operon, responsible for L-tryptophan (L-Trp) transport and catabolism, is regulated by L-Trp–directed translation arrest and the ribosome arresting peptide TnaC. The function of TnaC relies on conserved residues distributed throughout the peptide, which are involved in forming an L-Trp binding site at the ribosome exit tunnel and inhibiting the ribosome function. We aimed to understand whether nonconserved amino acids surrounding these critical conserved residues play a functional role in TnaC-mediated ribosome arrest. We have isolated two intragenic suppressor mutations that restore arrest function of TnaC mutants; one of these mutations is located near the L-Trp binding site, while the other mutation is located near the ribosome active site. We used reporter gene fusions to show that both suppressor mutations have similar effects on TnaC mutants at the conserved residues involved in forming a free L-Trp binding site. However, they diverge in suppressing loss-of-function mutations in a conserved TnaC residue at the ribosome active site. With ribosome toeprinting assays, we determined that both suppressor mutations generate TnaC peptides, which are highly sensitive to L-Trp. Puromycin-challenge assays with isolated arrested ribosomes indicate that both TnaC suppressor mutants are resistant to peptidyl-tRNA cleavage by puromycin in the presence of L-Trp; however, they differ in their resistance to puromycin in the absence of L-Trp. We propose that the TnaC peptide two functionally distinct segments, a sensor domain and a stalling domain, and that the functional versatility of these domains is fine-tuned by the nature of their surrounding nonconserved residues.

Ribosome arresting peptides (RAPs) are nascent polypeptides that act in *cis* on the translating ribosome to control the expression of genes by inducing ribosome arrest during translation elongation or termination. RAPs commonly sense external forces or low molecular weight compounds in the environment that spatially and temporally contribute to the expression of genes. RAPs such as SecM (1) that sense external forces on the ribosome are typically large, because these nascent peptides have a domain that functions outside of the ribosome. In contrast, those that sense small molecules inside of the ribosome, such as TnaC are smaller (2, 3). Typically, larger RAPs interact with cellular factors that can control their capacity for arresting ribosomes (4, 5, 6). Because of their size and proximity to ribosomal components, large RAPs clearly show two structural domains, a sensor domain and an arresting domain. At the moment of the arrest for the large RAPs, the sensor domain is located outside the ribosome exit tunnel (4, 5, 6), whereas the arresting domain remains inside the tunnel (5, 6, 7). The short RAPs currently characterized interact with the compounds that they sense by using the ribosome exit tunnel as a binding surface (8). For these short RAPs, it has been determined that conserved amino acid residues are necessary to induce arrest (9) by either directly binding the effector molecule or by acting at the peptidyl-transferase center (PTC) during ribosome arrest (2, 3, 10, 11). However, because the size of short RAPs ranges from only a few to a couple of dozen amino acids, as in the case of TnaC, it has remained unclear whether short RAPs are constituted by the two independent sensor and stalling domains, as it has been observed with larger RAPs.

In *Escherichia coli*, extracellular free L-tryptophan (L-Trp) is transported (12) and catabolized into indole, pyruvate, and ammonia (13) by proteins encoded by the *tnaCAB* operon (*tna*) (14). The *E. coli tna* operon’s transcript is organized into two functional segments: the first segment is comprised of a 5′-regulatory leader region containing the *tnaC* RAP gene and a *rut* site that serves as a binding site for the Rho transcription termination factor. The second segment contains the *tna* structural genes, *tnaA* and *tnaB*, which encode the L-Trp catabolic enzyme tryptophanase and an L-Trp transporter, respectively (15, 16). Transcription initiation is controlled by catabolite repression (17), while the continuation of transcription into the structural genes is regulated by nascent TnaC peptides through an L-Trp–dependent antitermination mechanism (18, 19). Under glucose-limiting conditions, transcription initiates at the *tna* promoter and continues until reaching intrinsic pause sites found within the untranslated spacer region (20). The paused polymerase permits translation of *tnaC* to begin and serves to synchronize the transcription of the structural genes with the translation of the leader region (20). Transcriptional termination at the 5′-regulatory leader region of the *tna* operon depends on changes in L-Trp concentrations that are sensed by the TnaC RAP within the ribosome. During translation, TnaC monitors for these fluctuations, by folding into two α-helical domains. Formation of the helical domains allows TnaC to compact and form an L-Trp–specific binding site (2, 3). In conditions of low L-Trp, the *tnaC* UGA stop codon enters the translating ribosome A-site where release factor 2 (RF2) induces hydrolysis of the resident TnaC-peptidyl-tRNA^Pro^, resulting in translation termination (18). The separation of the two ribosomal subunits then exposes the *rut* site that is positioned in the untranslated spacer immediately adjacent the *tnaC* stop codon (21), and Rho-dependent transcription termination (attenuation) ensues before transcribing the downstream catabolic genes (21). Alternatively, when L-Trp is in abundance, the compacted TnaC nascent chain will bind L-Trp and block RF2-mediated hydrolysis of the resident TnaC-peptidyl-tRNA^Pro^, leaving the arrested ribosome tethered to the mRNA (18, 22). The arrested ribosome blocks access of Rho to the *rut* site and transcription continues downstream expressing the structural genes (21).

TnaC contains four highly conserved amino acid residues that are critical for the induction of ribosome arrest by free L-Trp, namely W12, D16, I19, and P24, and mutations at these residues abolish L-Trp–dependent induction (16, 23, 24). A 2.6 Å cryo-EM structure of the TnaC-tRNA^Pro^-ribosome arrested complex identified W12 and D16 residues near the narrow region of the ribosome exit tunnel (known as constriction region) to be involved in binding a single free L-Trp molecule in conjunction with 23S rRNA nucleotides and the K90 residue of the ribosomal uL22 protein (2, 3). The uL22 K90 residue was previously identified by mutational changes to be important for L-Trp–induced expression of the *tna* operon (22, 25). This structure shows that TnaC folds into two distinct short α-helices connected by a hinge-like configuration with the W12 residue encompassed by the first α-helical structure (α_1_) at the constriction region. The second α-helix (α_2_) is close to the PTC and holds the I19 and P24 residues. The hinge is centered on the side chain of the I19 residue and positions the D16 residue at the top of the binding site. This unique structural distribution has not been observed in currently known structures of other large or short RAPs (Fig. 1) and suggests that TnaC might be structured with two independent functional domains.

**Figure 1:**
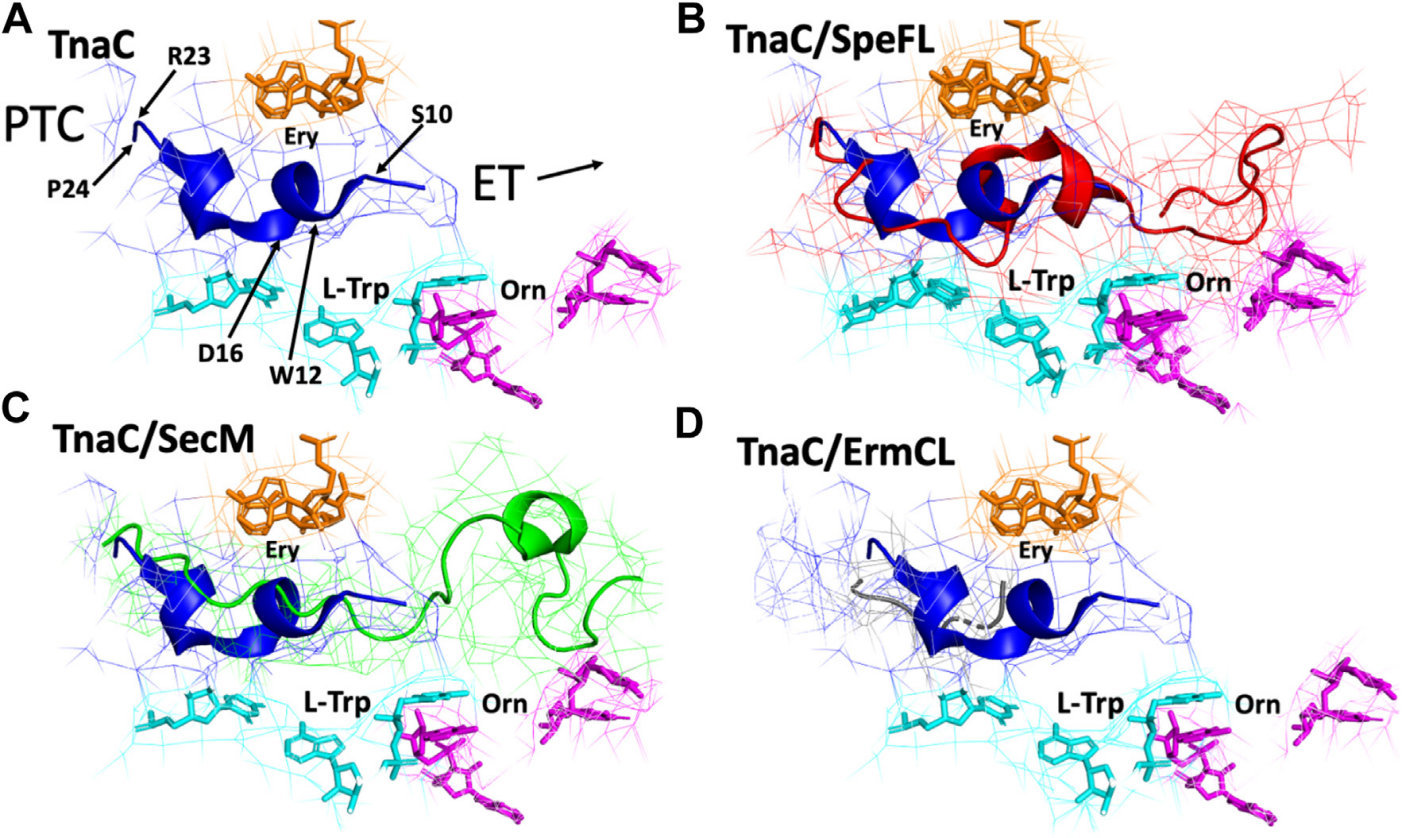
**Comparison of the structure of the TnaC peptide with other RAPs within the ribosome exit tunnel.***A*, predicted structure of nascent TnaC (*blue ribbon*) RAP. Positions of the PTC, exit tunnel (ET) direction, and TnaC residues studied in this work are indicated with *arrows*. 23S rRNA nucleotides involved in binding L-Trp (*cyan*) (2), erythromycin (*orange*) (33), or L-ornithine (*magenta*) (10) are shown for reference. *B*–*D*, nascent SpeFL (*B*, *red ribbon*), SecM (*C*, *green ribbon*), or ErmCL (*D*, *gray ribbon*) RAPs’ structures superimposed with TnaC. Models were made using PyMOL (https://pymol.org/2/) and the following PDB files: 701A (TnaC) (2), 6TC3 (SpeFL) (10), 6YS3 (SecM) (33), and 3J7Z (ErmCL) (34). L-Trp, L-tryptophan; PTC, peptidyl-transferase center; RAP, ribosome arresting peptide.

In this report, we compared the ability of several TnaC mutant peptides to induce ribosome arrest in response to L-Trp. Using reporter genes, we tested *in vivo* the suppressor capacity of two changes at nonconserved residues located in the α_1_ and α_2_ TnaC helixes, S10P and R23H, respectively. We tested the effects of these mutations to suppress loss-of-function (LOF) mutations at conserved residues W12, D16, and P24. Furthermore, we evaluated the L-Trp sensitivity *in vitro* of TnaC peptides containing either S10P or R23H. We show in this work that the nature of nonconserved residues in TnaC affects its capacity to sense L-Trp and arrest ribosomes during translation through two independent functional domains.

## Results

### Isolation of intragenic suppressor mutations of LOF TnaC(D16E) peptides at nonconserved TnaC residues

We have shown previously that mutating the nonconserved TnaC R23 residue partially restores the capacity for inducing ribosome arrest by the LOF TnaC(D16E) mutant peptide in the presence of L-Trp (2). Changes at this residue (R23H and R23F) located nearby the PTC reduce the kinetics for accommodating puromycin and RF2 at the PTC, increasing the opportunity of L-Trp to bind and induce ribosome arrest (2). We tested whether changes at other nonconserved positions of TnaC would produce suppressor effects similar to those induced by R23 changes. We decided to continue isolating suppressors for the LOF TnaC(D16E) mutant peptide through the strategy used previously (2). We identify a new suppressor mutation at the TnaC S10 residue, S10P. The TnaC S10 residue is positioned at the exit tunnel near the L-Trp binding site, far from the PTC (2). Our results showed that the S10P change partially suppressed the D16E noninducible phenotype. As seen in Table 1, the *tnaA’-’lacZ* reporter construct controlled by the *tnaC*(D16E/S10P) mutant in cells grown in the presence of L-Trp showed ∼10-fold higher β-galactosidase (β-gal) levels than those levels observed with the reporter gene controlled by the *tnaC*(D16E) mutant (compare row 5 with row 3, third column). In the absence of added L-Trp, the *tnaC*(D16E/S10P) reporter expressed ∼4-fold higher β-gal levels than the *tnaC*(D16E) reporter (Table 1 compare row 5 with row 3, second column). The *tnaC*(S10P) mutant alone did not affect the expression of the reporter gene in the presence of L-Trp; that is, we did not observed differences in the expression between the reporter constructs with *tnaC*(WT) and *tnaC*(S10P) (Table 1, compare row 4 with row 2, third column). However, the *tnaC*(S10P) mutant had a profound effect on the reporter gene expression when cells were cultured in the absence of L-Trp. The reporter gene with the *tnaC*(S10P) variant showed in absence of L-Trp ∼20-fold higher β-gal levels than its counterpart with the WT *tnaC* variant (Table 1, compare row 4 with row 2, second column). These data show that the S10P change by itself substantially increased expression of the *tnaA’-’lacZ* reporter gene in the absence of exogenously added L-Trp.

**Table 1:** S10P and R23H are *cis*-acting mutations that suppress the LOF D16E mutation

Strain	β-gal activity (MU)^a^		Induction ratio (+Trp/-Trp)^b^
	-Trp	+Trp	
WT	57 ± 2	2175 ± 274	38.2
D16E	38 ± 11	68 ± 25	1.8
S10P	1312 ± 290	2506 ± 244	1.9
S10P/D16E	138 ± 14	568 ± 137	4.1
WT (ΔAUG)	7 ± 1	7 ± 0	1.0
D16E (ΔAUG)	7 ± 1	7 ± 0	1.0
S10P (ΔAUG)	8 ± 0	8 ± 2	1.0
S10P/D16E (ΔAUG)	10 ± 0	9 ± 1	0.9

Abbreviations: ACH, casein acid hydrolyzed; β-gal, β-galactosidase; LOF, loss-of-function; L-Trp, L-tryptophan; M9-MM, M9 minimal medium; MU, Miller units.

a Cultures of *Escherichia coli* bacterial strains AW153 (WT), AW821 (D16E), AW888 (S10P), AW909 (D16E/S10P), AW643 (WT(ΔAUG)), AW940 (S10P (ΔAUG)), AW946 (D16E (ΔAUG)), and AW949 (S10P/D16E (ΔAUG)) were grown in M9-MM plus 0.2% glycerol, 0.05% ACH, 0.01% vitamin B1, with (+Trp) or without (-Trp) 100 μg/ml L-Trp. β-gal assays were performed in three independent experiments.

b Induction ratio of values for cultures grown with L-Trp (+Trp) and those cultured without L-Trp (-Trp).

At least two different mechanisms might explain the high basal level expression of *lacZ* observed with the S10P mutant in absence of L-Trp. One possibility is that changes in the S10 codon affect *tnaC* mRNA secondary structure leading to inefficient transcription termination of the *tnaC* regulatory region. A second possibility is that the TnaC(S10P) mutant RAP alone induces ribosome arrest at the low L-Trp levels found *in vivo* in the absence of exogenously added L-Trp. To discern between these possible scenarios, we tested *in vivo* the expression of our reporter gene with WT and mutant *tnaC* variants with or without their *tnaC*-AUG start codon. If the mutant RAP causes the high basal levels of the reporter gene, then in the absence of its translation (absence of the start codon), premature transcription termination will occur in the reporter gene leading to a drastic decrease in -gal expression in either the presence or absence of L-Trp. As expected, the absence of translation of WT *tnaC* dramatically decreased the expression of *lacZ* both in the presence or absence of additional L-Trp (Table 1, compare row 6 with row 2). Likewise, the expression of the reporter gene was also dependent on the translation of the TnaC(S10P) (Table 1, compare row 8 with row 4) and TnaC(D16E/S10P) (Table 1, compare row 9 with row 5) mutant variants. The data obtained with *tnaC*(S10P) mutant variants is similar to those observed with *tnaC*(R23H) mutants (Table S3) (2). These results indicate that the effects of the S10P change are dependent on translation of the mutant TnaC peptide and do not reflect effects exerted by structural changes in the RNA sequence affecting transcription termination. Taken together, these data indicate that the S10P change could promote similar effects at the PTC of the TnaC–ribosome complex as observed with the R23 variants (2). Alternatively, the S10P mutation could alter the sensitivity of TnaC for L-Trp without affecting the PTC.

### Effects of the S10P and R23H mutational changes on the TnaC RAP function assayed by regulation of antibiotic resistance

**Figure 2:**
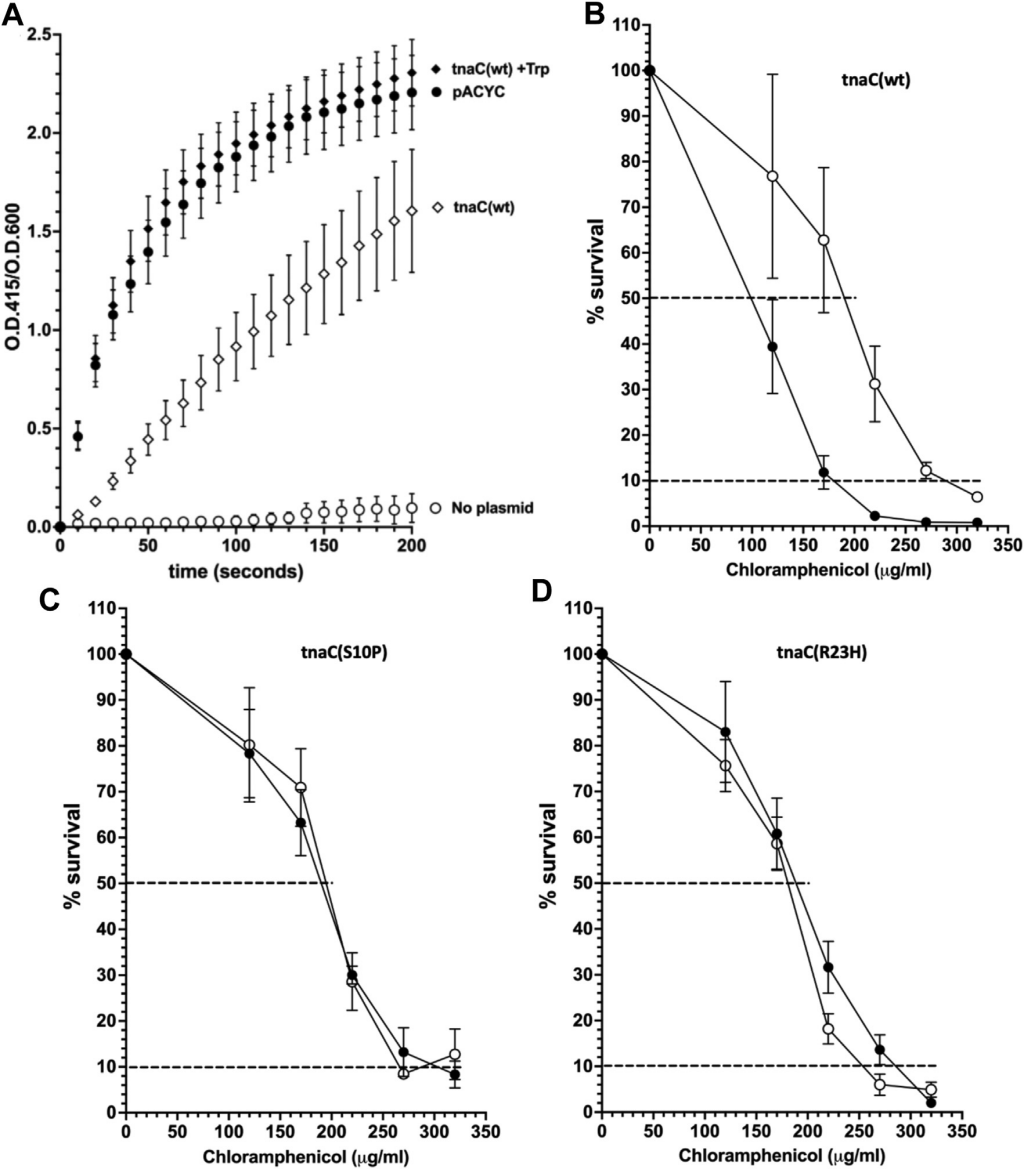
**CAT expression and survival of bacterial cells containing *tnaC tnaA’-‘camR* reporter gene fusions.***A*, plot showing a time course of the accumulation of products resulting from CAT activity in cell-free extracts normalized to cell density (*A*_415_/*A*_600_). CAT activity assays were performed using extracts obtained from cells containing or lacking the indicated constructs (Experimental procedures). In the case of cells with the *tnaC tnaA’-‘camR* construct (*tnaC*), cultures were grown with (+Trp) or without 25 μg/ml of 1-MTrp. Bars indicate the SEM of two independent experiments. *B*–*D*, plots showing the % survival of bacterial cells with plasmid constructs containing (*B*) *tnaC* WT, (*C*) *tnaC*(S10P), and (*D*) *tnaC*(R23H) genes. Cultures induced for an hour with (*open symbols*) or without (*close symbols*) 20 μg/ml of 1-MTrp were challenged with several chloramphenicol concentrations (Experimental procedures). *Dotted lines* indicate the chloramphenicol concentration ranges for which cell surviving was 50% and 10%. Bars indicate the SE of the ratio of means values for six independent experiments. 1MTrp, 1-methy-L-Trp; CAT, chloramphenicol acetyl-transferase.

To confirm and extend the findings obtained using *lacZ* reporter assays, we generated a plasmid construct to analyze cell survival based on TnaC expression. A *tnaC tnaA’-‘camR* reporter construct was produced by replacing the transcriptional regulatory region of the chloramphenicol resistance *camR* gene encoding chloramphenicol acetyl-transferase (CAT) in pACYC184 with the 5’-regulatory region of the *tna* operon (Experimental procedures). Thus, with this reporter, CAT protein expression is under the control of TnaC and free L-Trp. Initially, we monitored *camR* expression of our constructs using an enzymatic assay that measures the concentration of free sulfhydryls that result from CAT activity (Experimental procedures). Cell lysate from cultures grown in the presence of L-Trp produced CAT rate of reaction of 0.037 U, while extracts from cells grown without L-Trp produced a rate of reaction = 0.01 U. These results indicate a ≈3.7-fold increase in CAT activity of cells grown with L-Trp compared to those cells that were not cultured with exogenous L-Trp (Fig. 2*A*, compare closed diamonds with open diamonds, respectively). We concluded that WT TnaC peptides induce *camR* expression in the presence of L-Trp. Levels of CAT expression in cells exposed to L-Trp was similar to that in cells containing the natural *camR* promoter (pACYC plasmid) (Fig. 2*A*, compare closed circles with closed diamonds), suggesting that both regulatory regions *tna* and *camR* have similar transcriptional and translational efficiencies. In parallel to the CAT enzymatic assays, we also performed, cell growth challenges with chloramphenicol (Cm) to calculate the inhibitory concentration for reducing 50% (IC_50_) and 90% (IC_90_) of growth in cultures of *E. coli* harboring the *tnaC tnaA’-‘camR* reporter (Experimental procedures). Corresponding to what was observed with the CAT enzymatic assays, we observed that cell cultures containing WT *tnaC* reporter plasmids were more resistant to Cm in the presence of L-Trp than in its absence (Fig. 2*B* and Table 2, row 2). These data show that the *tnaC tnaA’-‘camR* reporter gene construct can be used to analyze and quantify the L-Trp sensing capacity of the 5’-*tna* regulatory region by determining Cm-IC values of cell growth and CAT activity levels.

**Table 2:** IC_50_ and IC_90_ values of cell growth of bacteria with *tnaC tnaA’-‘camR* reporter gene fusion variants

*tnaC* gene	-Trp		+Trp^a^	
	IC_50_^b^	IC_90_	IC_50_	IC_90_
wt	<120	170<	170<IC<220	270<
S10P	170<IC<220	320<	170<IC<220	320<
R23H	170<IC<220	320<	170<IC<220	320<
D16 changes				
D16E	<120	<120	<120	<120
S10P/D16E	120<IC<170	220<	120<IC<170	220<
D16E/R23H	270<IC<320	320<	270<IC<320	320<
D16K	<120	<120	<120	<120
S10P/D16K	120<IC<170	220<	120<IC<170	220<
D16K/R23H	270<IC<320	320<	270<IC<320	320<
D16A	<120	<120	<120	<120
S10P/D16A	170<IC<220	270<	170<IC<220	270<
D16A/R23H	270<IC<320	320<	270<IC<320	320<
W12 changes				
W12R	<120	<120	<120	<120
S10P/W12R	120<IC<170	220**≤**	120<IC<170	220 **≤**
W12R/R23H	**≤**320	320**≤**	**≤**320	320**≤**
W12L	<120	<120	<120	<120
S10P/W12L	120<IC<170	170<	120<IC<170	170<
W12L/R23H	**≤**320	320**≤**	**≤**320	320**≤**
W12A	<120	<120	<120	<120
S10P/W12A	<120	170<	<120	170<
W12A/R23H	<120	<120	<120	<120
P24 changes				
P24C	<120	170	<120	170
S10P/P24C	<120	170	<120	170
R23H/P24C	170<IC<220	270	270<IC<320	320<

Abbreviations: 1MTrp, 1-methy-L-Trp; M9-MM, M9 minimal medium.

a Cultures were grown in M9-MM with (+Trp) or without (-Trp) 20 μM 1MTrp as indicated in Experimental procedures.

b IC values in μg/ml were estimated using the plots shown at Figure 1 and supporting information (Figs. 1, S3 and S5).

We used *tnaC tnaA’-‘camR* reporters to test the impact of *tnaC* variants containing changes at the *tnaC* S10 and R23 codon positions. In the absence of L-Trp, cells harboring constructs with either *tnaC*(S10P) or *tnaC*(R23H) mutant genes were more resistant to Cm than those cells containing the WT *tnaC* construct (Table 2 compare row 2 with rows 3 and 4). As expected, in the absence of L-Trp the IC_50_ and IC_90_ values for cells with either TnaC(S10P) or TnaC(R23H) peptides were similar to those observed with the addition of L-Trp (Fig. 2, *C* and *D*, compare open with closed circles; Table 2 row 3 and 4, compare columns 4 and 5 with 2 and 3). The results obtained from drug-challenge assay corresponded to the relative measurements of CAT activity obtained from cell-free extracts of these cultures (See Figs. S2 and S4), confirming that both S10P and R23H induce higher levels of expression of the *tnaA’-‘camR* gene even in the absence of added L-Trp *in vivo*.

### Effects of the S10P suppressor mutation on the LOF TnaC mutations that affect L-Trp binding site

The TnaC S10P and R23 mutations were initially isolated as suppressors of the LOF TnaC(D16E) mutant which was not inducible by L-Trp. Therefore, we wanted to determine if the S10P mutation could restore L-Trp–dependent induction to other LOF TnaC mutants. We place the *tnaC*(S10P) mutation into *tnaC tnaA’-‘camR* constructs containing mutations at either of two other sites with, W12 or D16, that by themselves result in the TnaC LOF phenotype. The substituted codons included amino acids with various sidechain arrangements among them we added variations in the number of carbons and charges. The double-mutants tested included S10P/W12R, S10P/W12L, S10P/W12A, S10P/D16E, S10P/D16K, and S10P/D16A. Cells with the constructs expressing TnaC(S10P/D16E) peptides resisted higher Cm concentrations with and without L-Trp than the cells encoding D16E peptides under either condition (Table 2, compare row 7 with row 6; Fig. S1). These results correspond to those obtained with the -gal assays using the *tnaC tnaA’-‘lacZ* reporter construct (Table 1). Similar results were also observed for cells containing construct sequences encoding TnaC(S10P/D16K) and TnaC(S10P/D16A), where both showed higher resistance to Cm in the presence and absence of L-Trp than their corresponding single mutation (D16X) (Table 2, compare row 10 with row 9, and row 13 with row 12 respectively; Fig. S1, *A*–*C*). The survival results corresponded with CAT enzymatic activity obtained using cell-free extracts of these cultures (Fig. S2, *A* and *B*). These data indicate that the S10P mutation can suppress the LOF phenotype that results from changes at the D16 residue. Consistent with the data obtained with the *tnaC tnaA’-‘lacZ* reporter construct (Table S4), the S10P mutation is more efficient at suppressing the LOF phenotype associated with the D16A mutant compared to D16E or D16K (Table 2, compare row 13 with rows 7 and 10). Cells expressing TnaC(S10P/W12R) resisted higher Cm concentrations than those with TnaC(W12R) with and without L-Trp (Table 2, compare row 17 with row 16; Fig. S1*D*). Similarly, when TnaC contained the W12L change, cells expressing TnaC(S10P/W12L) resisted higher Cm concentrations than those with TnaC(W12L) with or without L-Trp (Table 2, compare row 20 with row 19; Fig. S1*E*). In contrast to what we observed for the TnaC(D16A/S10P) mutant, S10P effects were less efficient in the TnaC(S10P/W12A) mutant, although the TnaC(S10P/W12A) mutant still increased the cells resistance to Cm when culture media was supplemented or not with L-Trp relative to TnaC(W12A) (Table 2, compare row 23 with row 22; Fig. S1*F*). Corresponding levels of CAT activity were obtained from cell-free extracts, where more activity was observed from cells with TnaC(S10P/W12R) and TnaC(S10P/W12L) than those with TnaC(S10P/W12A) cultured with (Fig. S2*C*) or without L-Trp (Fig. S2*D*). Overall, our data indicates that the S10P change can suppress the effects of LOF mutations on essential TnaC amino acid residues, and these effects are dependent on both the position of the conserved amino acid residue within the TnaC peptide and the nature of the substituted amino acid that generated the nonfunctional RAP.

### Functional consequences of the R23H suppressor mutation on the LOF of mutations at W12 and D16

We asked whether the R23H mutation had the same effects on LOF mutations at W12 and D16 that we observed with S10P. The R23H mutation was combined with the W12 and D16 mutations to produce the following combinations: D16E/R23H, D16A/R23H, and D16K/R23H; and W12R/R23H, W12L/R23H, and W12A/R23H. Similar to what was observed with the S10P double mutants, R23H suppressed the D16 LOF phenotypes of several mutations (Table 2 and Fig. S3). All double mutants D16E/R23H, D16K/R23H, and D16A/R23H demonstrated higher resistance to Cm than the corresponding single D16X mutants when the media was supplemented or not with L-Trp (Table 2 and Fig. S3, *A*–*C*). CAT activity measurements corresponded with the survival data (Fig. S4, *A* and *B*) and with data obtained also using *tnaC tnaA’-‘lacZ* reporter constructs (Table S5). For the W12 peptides, the R23H mutation suppressed the W12R and W12L changes where cells expressing either TnaC(W12R/R23H) or TnaC(W12L/R23) mutant variants were more resistant to Cm than their single W12X counterpart (Table 2 and Fig. S3, *D* and *E*), with corresponding impacts on CAT activity levels(Fig. S4, *C* and *D*). Interestingly, the R23H change did not suppress the LOF phenotype produced by the W12A change. Cells expressing the TnaC(W12A/R23H) double mutant were less resistant to Cm than cells expressing the single TnaC(W12A) mutant (Table 2, compare row 24 with row 22; Fig. S3*F*) nor did these cells produce more CAT enzyme (Fig. S4, *A*, *C* and *D*). These data indicate that the effect of the R23H change on LOF mutants, as seen previously with the S10P mutant, depends on the position of the mutated TnaC residues and chemical nature of the amino acid replacement.

### Suppression of changes at the tnaC P24 codon position

Finally, we tested the capacity of S10P and R23H to suppress the LOF phenotype resulting from a mutation at the conserved amino acid residue located at the PTC, P24. As seen in Table 2 and Fig. S5, the P24C mutation abolishes induction of reporter gene expression by L-Trp, and cells with this mutation are unable to survive in the presence of Cm either supplementing or not with L-Trp (Table 2 compare row 26 with row 2; Fig. S5*A*). These results were confirmed using CAT activity assays, where low levels of CAT activity were observed with the P24C mutant under the tested conditions (Fig. S6, *A* and *B*). Due to the proximity of the R23H mutation to the PTC, unlike the S10P, we hypothesized that the R23H mutation could at least partially restore L-Trp–dependent induction of TnaC peptides with the P24C change. Results from survival assays supported this idea and showed that cells expressing TnaC(R23H/P24C) peptide were more resistant to Cm than those cells expressing the TnaC(P24C) peptide when cultured with or without L-Trp (Table 2 compare row 28 with row 26; Fig. S5*B*). In contrast, cells expressing TnaC(S10P/P24C) were similarly resistant to Cm concentrations compared to TnaC(P24C) independent of added L-Trp (Table 2 compare row 27 with row 26; Fig. S5*A*). CAT activity assays were consistent with survival results (Fig. S6*B*). Overall, these data indicate that the R23H mutation, unlike S10P, can suppress the LOF phenotype associated with changes at the TnaC P24 residue.

### *In vitro* L-Trp sensitivity of the TnaC–ribosome complex with S10P and R23H changes

**Figure 3:**
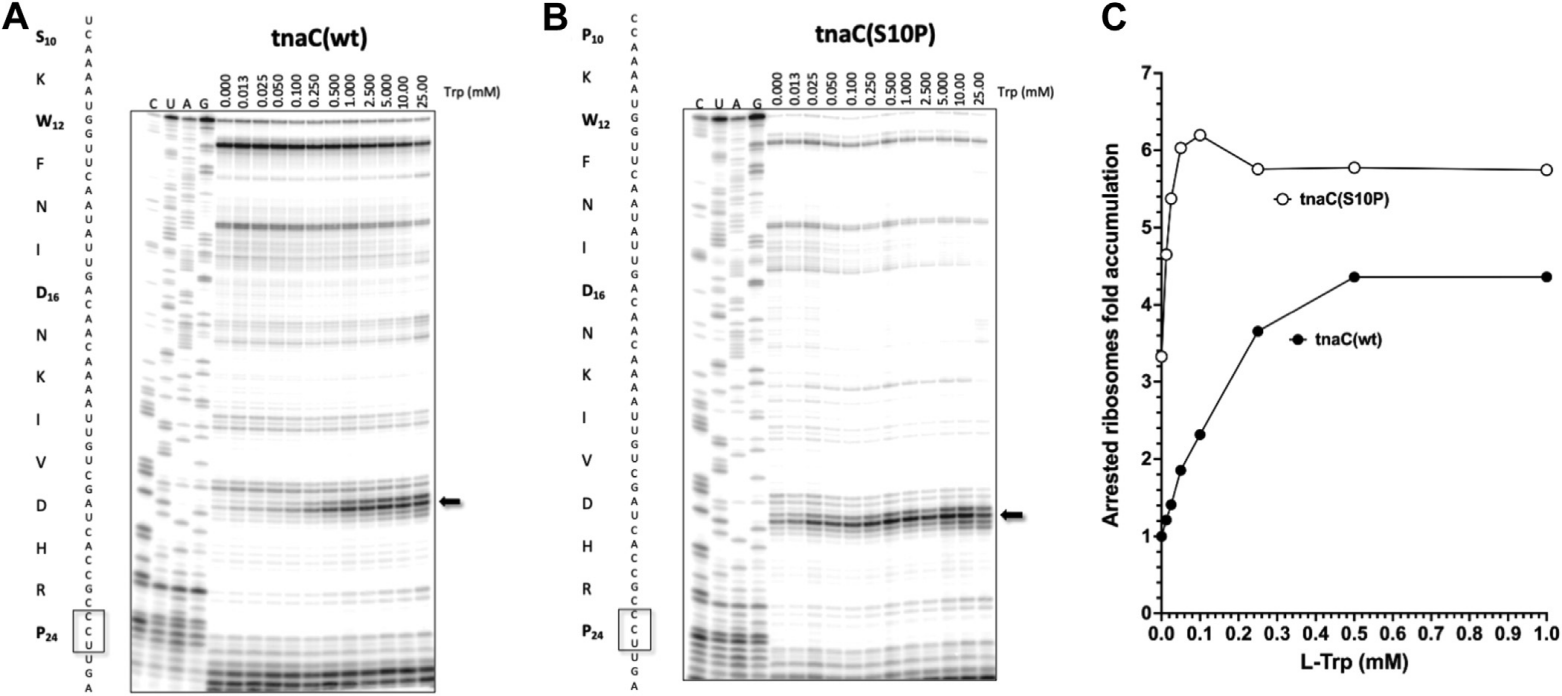
***In vitro* accumulation of *Escherichia coli* arrested ribosomes during *tnaC* expression assayed by toeprinting and autoradiography.***A*, WT *tnaC*(wt) and (*B*) mutant *tnaC*(S10P) PCR DNA fragments were used to detect arrested ribosomes over a series of reactions containing a shown range of L-Trp concentrations. *C*, plot representing fold accumulation of stalled ribosomes *versus* L-Trp concentrations. Each data point represents one independent experiment. L-Trp, L-tryptophan.

Toeprinting assays have previously been used to determine the sensitivity of TnaC–ribosome complexes to L-Trp by identifying the position of arrested ribosomes induced by L-Trp on *tnaC* mRNA sequences (2, 24). With this approach, we compared the sensitivity of ribosomal complexes containing either WT or S10P TnaC peptides to become arrested in response to L-Trp (Fig. 3). We observed, as expected, that ribosome arrest occurred at the position of the Pro-24 codon with both the WT and mutant S10P *tnaC* ORFs in response to different concentrations of L-Trp (Fig. 3, *A* and *B*). Quantification of the arrest signal at Pro-24 codon (Experimental procedures) showed that the TnaC(S10P) mutant is more sensitive to low concentrations (<0.25 mM) of L-Trp than the TnaC(WT) (Fig. 3*C*). These results indicate that stalling at the Pro-24 codon of the *tnaC*(S10P) mutant still requires the addition of L-Trp, but the sensitivity of the TnaC(S10P)–ribosomes complex toward L-Trp is higher than that of the WT TnaC peptide–ribosome complex.

**Figure 4:**
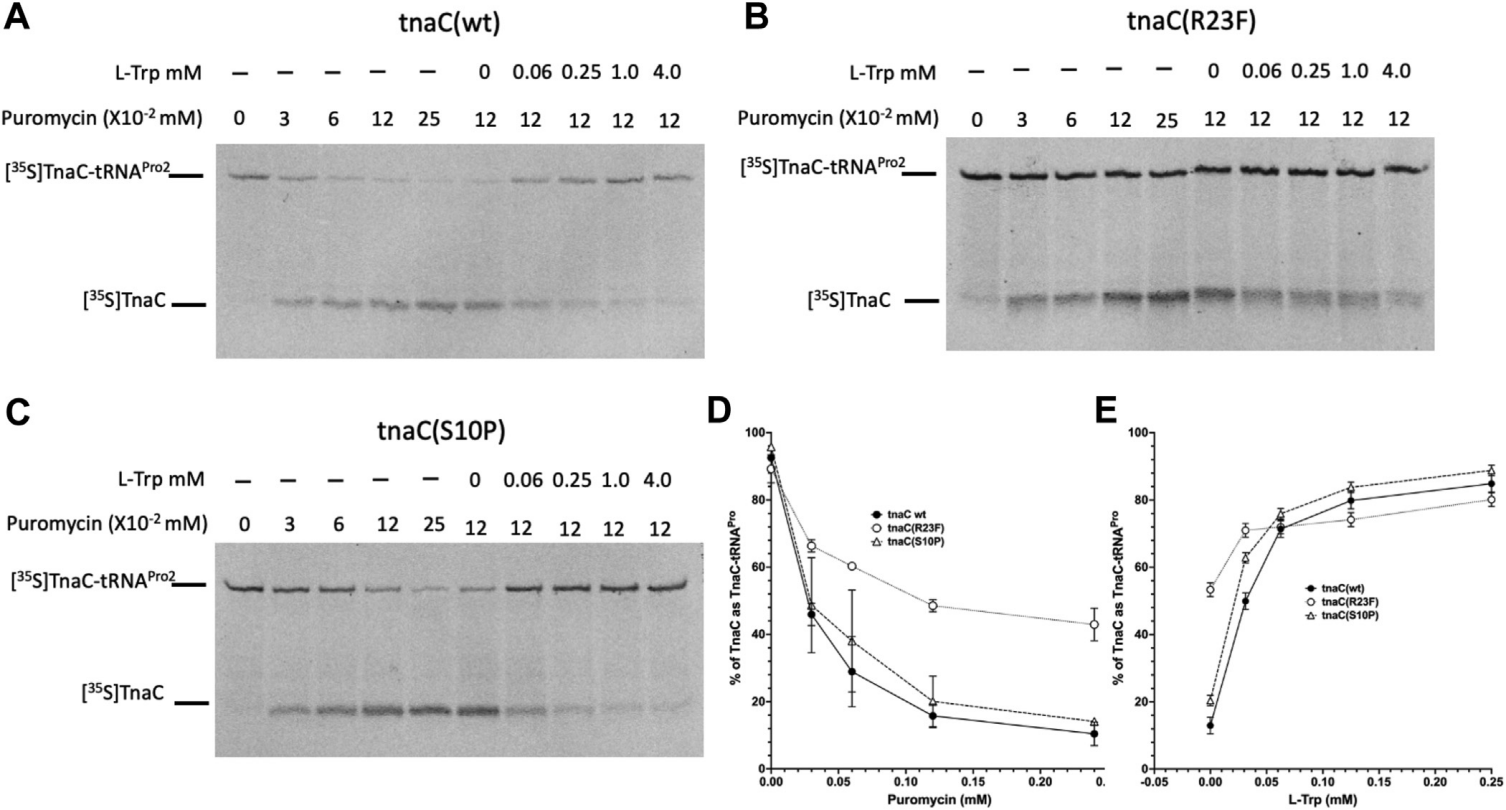
**Ability of arrested ribosomes to transfer the TnaC peptide from the TnaC-peptidyl-tRNA**^**Pro**^**to puromycin.***A*–*C*, autoradiography showing the resolved products of puromycin resistance assays. Isolated ribosomal complexes containing [^35^S]-Met labeled TnaC-tRNA^Pro^ were obtained from *in vitro* cell-free reactions with either WT, S10P, or R23F *tnaC* mRNA. The complexes were incubated with (+) or without (−) several concentrations of L-Trp to allow ligand binding and later incubated with several concentrations of puromycin. *Arrows* indicate the position of the [^35^S]-Met-TnaC-tRNA^Pro^ and [^35^S]-Met-TnaC-puromycin (TnaC) molecules. *D* and *E*, plots showing % of [^35^S]-Met-TnaC as [^35^S]-Met-TnaC-tRNA^Pro^ in the WT, R23F, and S10P mutant complexes after treatment with puromycin. Bars indicate the SEM of two independent experiments. [^35^S]-Met, [^35^S]-methionine.

Formation of the TnaC(S10P)-stalled ribosome complex at low concentrations of L-Trp could be due to disruption of the PTC activities imposed by the mutant nascent peptide even in the absence of L-Trp, as we had previously observed for complexes formed with TnaC(R23F/H) mutant variants (2). To evaluate this possibility, we monitored the ability of the ribosome to transfer the WT and mutant versions of TnaC from the TnaC-tRNA^Pro^ molecule to puromycin in the presence or absolute absence of L-Trp (26). Isolated TnaC–ribosome complexes obtained from *in vitro* reactions with either WT or mutant *tnaC* mRNAs were challenged with puromycin and the reaction products were resolved in electrophoresis gels (Experimental procedures). Results can be seen at Figure 4, *A*–*C*. In the absence of L-Trp, reactions with TnaC(WT) and TnaC (S10P)–tRNA^Pro^ complexes were similarly sensitive to puromycin cleavage, whereas those with TnaC(R23F)–tRNA^Pro^ complexes were more resistant to puromycin cleavage (Fig. 4*D*). These results demonstrate that the S10P change is not affecting the ribosome active site, while R23F affects this site. We confirmed that these isolated TnaC–ribosome complexes all remain responsive to L-Trp and show that L-Trp addition protected TnaC-peptidyl-tRNA^Pro^ from the cleavage action of puromycin in all WT TnaC and both the S10P and R23F variants (Fig. 4*E*). However, TnaC-tRNA^Pro^ molecules with either S10P or R23F mutations, showed increased resistance to puromycin cleavage at lower (<0.25 mM) L-Trp concentrations than that observed with the WT version (Fig. 4*E*). These last set of data indicate that the S10P mutation makes the TnaC RAP more sensitive to L-Trp *in vitro* than the WT TnaC RAP.

## Discussion

In this work, we studied the effects of two intragenic mutations that partially restore L-Trp–dependent induction in LOF mutant variants of the TnaC RAP (Tables 1 and 2, S3–S5). Using a reporter gene construct that links the expression of CAT to the *tna* regulatory region (Fig. 2), we observed that two TnaC(S10P) and TnaC(R23H) mutants increase the sensitivity of the TnaC RAP for sensing L-Trp compared to WT TnaC peptide (Tables 1 and 2, S3–S5). We used this construct to analyze the suppressor effects of S10P and R23H mutations on LOF TnaC mutations. We observed that the S10P replacement can partially suppress the LOF phenotype associated with D16 and W12 mutations, but not P24 mutations (Table 2) mutations; whereas R23H can partially suppress almost all LOF mutations tested at the D16, W12, and P24 positions, with the exception of W12A (Table 2). We show that the nascent TnaC(S10P) peptide induces accumulation of ribosome arrest at lower L-Trp concentrations than WT TnaC peptides (Fig. 3), and that, in contrast to the R23F, S10P does not have a general effect on the action of puromycin *in vitro* but instead increases the capacity of L-Trp to block puromycin (Fig. 4). Additionally, our data suggests that nascent TnaC peptides containing S10P do not affect the activity of the PTC in TnaC-arrested ribosome complexes in the manner of TnaC(R23F) peptides.

**Figure 5:**
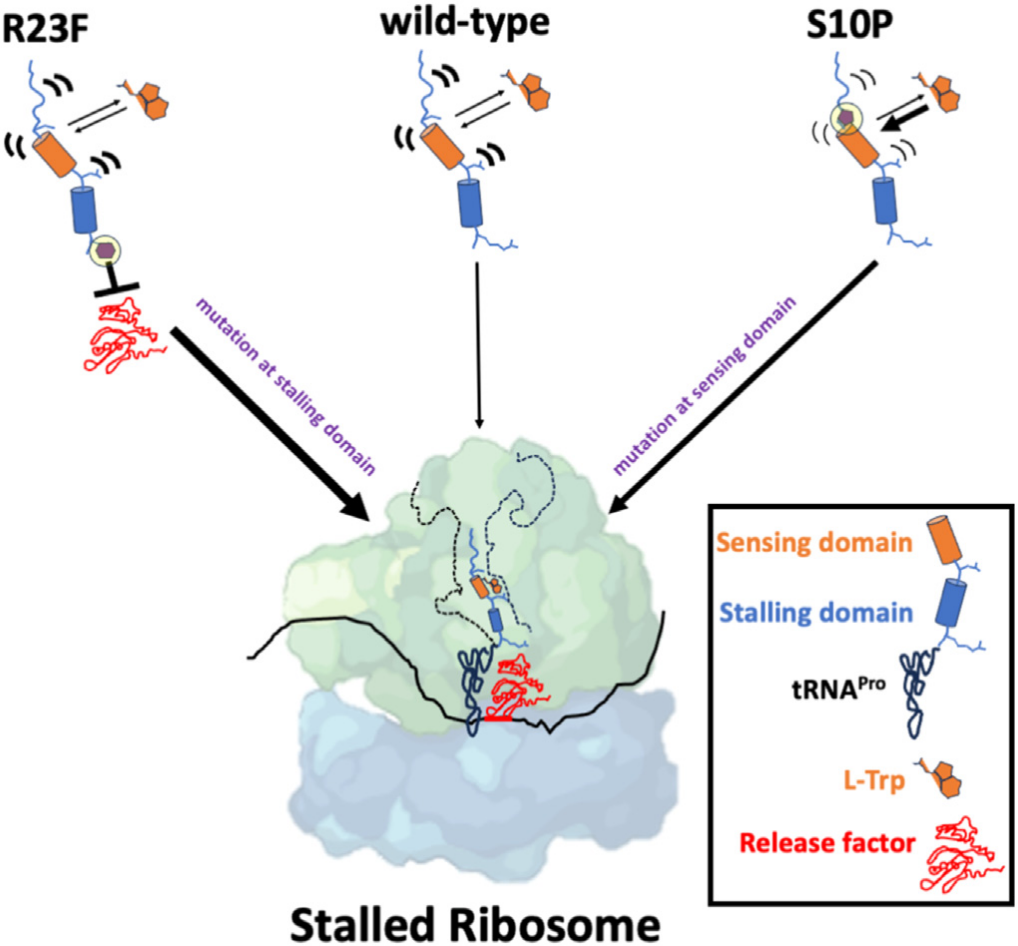
**Proposed model of ribosome stalling induced by TnaC mutants and L-Trp.** Changes in the surrounding residues of the TnaC functional domains increase the efficiency of production of stalled ribosomes containing TnaC-tRNA^Pro^ molecules and release factors. WT TnaC peptides bind with L-Trp inside the tunnel through interactions with the peptide’s sensor domain, blocking functional accommodation of the release factor through its stalling domain at the PTC. The R23F change at the TnaC’s stalling domain reduces the kinetics of release factor functional accommodation (*bold line*), allowing L-Trp to bind at lower concentrations. On the other hand, the S10P change at the TnaC’s sensing domain increases the binding kinetics of L-Trp, allowing L-Trp to compete with the release factor functional accommodation. The *thickness of arrows and curved lines* indicates a tendency toward interactions or molecular states. Figures were made based on previous cryo-EM structures (2). L-Trp, L-tryptophan; PTC, peptidyl-transferase center.

Our results support the idea that induction of ribosome arrest by the TnaC peptide requires two distinct domains called the sensor and stalling domains as previously suggested (2, 27), with S10P affecting the sensor domain and R23F affecting the stalling domain (Fig. 5). The sensor domain, used to bind free L-Trp within the tunnel, contains the conserved TnaC residues W12 and D16, and, to a lesser extent, neighboring less-conserved residues such as S10 (2). The stalling domain, which is constituted by P24 and neighboring residues such as R23, acts at the PTC to reduce the binding kinetics of active molecules such as puromycin and RF2 at the ribosome active site (2).

Three TnaC mutations S10P, R23H, and R23F have been identified that increase the sensitivity of TnaC for L-Trp. R23F was first identified in TnaC peptides that could arrest ribosomes during the elongation phase (23, 28) while S10P and R23H mutations were isolated as a part of this work to function as suppressor mutations that restore induction of noninducible D16E peptides. The R23F mutation produces a stable ribosome-arresting complex that was exploited to obtain a high-resolution 2.4 Å cryo-EM model of the TnaC-arrested ribosome bound by L-Trp (2). Changes at the R23 TnaC residue reduce the susceptibility of TnaC toward puromycin transfer independent of L-Trp (Fig. 4*D*) (2). Because the R23 amino acid residue is close to the PTC, we expected that changes at this position could reduce the kinetics of puromycin and RF2 action at the PTC, allowing the TnaC–ribosome complex to interact with L-Trp with a higher efficiency (Fig. 5). This could explain the suppressor effects that R23H has on the LOF P24C mutation (Table 2). As previously proposed, we suggest that the presence of a His or Phe residue at the R23 position clashes with the Q252 residue of RF2 in the PTC A-site (2, 3, 29) and reduces the rate of accommodation for RF2 (30). However, we cannot eliminate the possibility that an R23H/P24C double mutation may also affect the binding of Rho, as suggested previously (31).

Our current TnaC-ribosome structure shows the S10 residue near the L-Trp binding site just beyond the constriction region (2, 3). Therefore it is unlikely that the S10P residue change physically obstructs proper accommodation of molecules in the A-site, in contrast to R23F/H changes, and thus must exert its forces in a different manner. We have shown that S10P increases L-Trp sensitivity without affecting the PTC (Fig. 4) and hypothesize that the presence of a Pro residue at the 10th TnaC position increases the affinity of the binding site for L-Trp. Based on the density map obtained with the 2.4 Å cryo-EM structure, no interactions between the S10 residue were observed with free L-Trp, uL22 protein, or 23S rRNA nucleotides. Furthermore, S10 is not a conserved residue in the family of TnaC peptides (23). However, experiments using TnaC peptides with N-terminal truncations indicate that at least two residues should precede the W12 residues to maintain a functional peptide (2). This observation implies that W12 depends on the S10 residue for spatial distribution within the ribosome exit tunnel and that a proline residue at this position might increase the chances for W12 to establish a functional conformation (Fig. 5). Replacing the W12 residue with a small amino acid, such as Ala, reduces the ability of S10P to suppress the LOF phenotype (Table 2), indicating that the increased affinity for binding L-Trp is dependent on the size of the 12th residue. In our view, this suggests that formation of the binding site able to hold free L-Trp, as observed by cryo-EM (2), requires a long-chain or bulky amino acid residue at TnaC position 12. In addition, as previously suggested (32), the large amino acid size of TnaC position 12 may also favor the transmission of structural information through the TnaC peptide to the PTC once it interacts with free L-Trp. These last ideas are supported by the lack of suppressor activity observed with the R23H change on the W12A mutation (Table 2). That is, despite the action of the R23H mutation to reduce activity at the PTC, TnaC(R23H) peptides must still rely on the binding of free L-Trp to obtain maximum arresting capacity (Fig. 5).

Contrary to the results obtained with W12 changes, our data suggest that long-chain amino acid residues at the D16 position, such as Lys, hampers the ability of either S10P or R23H mutations to suppress the LOF phenotypes (Table 2). The D16 residue interacts with the U2609 23S rRNA nucleotide residue at the peptide exit tunnel (2, 3) and is important for L-Trp induction of the *tna* operon (25). D16 is in a region of the TnaC peptide between two alpha helices that form a 110˚ hinge which permits the formation of the TnaC arresting configuration (2). D16 is important for the action of otherwise WT TnaC peptides (Table 2) (23). However, when TnaC peptides contain either S10P or R23H, the residue identity at position 16 becomes less critical, as small residues besides Asp, such as Ala, allow TnaC(S10P or R23H) function (Table 2). We suggest that the small size of the Ala residue at position 16 serves as a placeholder within the peptide for the proper positioning of essential rRNA residues of the tunnel that are encompassed in either the sensor or stalling functional domains. Structural changes in the TnaC peptide produced by S10P and R23H might shift the position of the D16 residue inside the exit tunnel to be closer to the 23S rRNA residues. Substituting position 16 of TnaC with a small amino acid residue perhaps still assists in the formation of the hinge required for the TnaC arresting configuration.

The work presented here shows how mutations in nonconserved residues can be used to modulate the responsiveness of RAPs. The two mutations, S10P and R23H, both increase the sensitivity of TnaC for sensing L-Trp and partially suppress the LOF phenotype associated with some (but not all) mutations that abolish induction by L-Trp. We propose that both S10P and R23H suppressors create changes in the overall structure of the TnaC peptide that abrogates the necessity of the essential residues to enable folding into a functional configuration. These data support the idea that nonconserved residues also can significantly modulate the functions of this arrest peptide. Our observations on the versatility of TnaC peptide variants to induce ribosome arrest suggest that the sequences of the regulatory arrest peptides from different bacteria have been evolutionarily selected to adjust expression of the *tna* operon according to the L-Trp levels in the environment. Future biochemical and structural studies may determine whether TnaC-regulated *tna* operons from other bacterial strains respond to specific L-Trp concentrations and if these TnaC peptide variants could sense other metabolites, which may illuminate how to rationally design new RAPs able to respond to small molecules of interest.

## Experimental procedures

### Strains and plasmids

Plasmids used in this work are shown in Table S1. Bacteria containing a *tnaC tnaA’-‘lacZ* chromosomal single copy were constructed as indicated previously (25). A *tnaC tnaA’-‘camR* plasmid construct was made as follows: unique restriction sites (*PstI* and *PvuI*) were inserted 24 and 6 base pairs, respectively, upstream of the ATG codon of the *camR* resistance gene of the pACYC184 plasmid using the Q5 site-directed mutagenesis kit (New England Biolabs, Cat. no. E0554S). Restriction sites were inserted using the following oligonucleotides 5′-GTGATTTTTTTCTCCATTTTAGCCGATCGTTCCTT-3′ and 5′-AGCTCCCTGCAGTGAAAATCTCGATAACTCAAAAAATAC-3′. The *tna* leader region containing the *tna* promoter, *tnaC* gene, and the intergenic region between *tnaC* and *tnaA* through the seventh codon of *tnaA* was amplified by PCR using chromosomal DNA obtained from a W3110 (*F*^*-*^ *λ*^*-*^ *rph-1 IN*(*rrnD*, *rrnE*), *kdgR*^-^) strain using the following primers 5′-CTG**CTGCAG**GCTTCTGTATTGGTAAGTAACC and 5′-GCGT**CGATCG**GAGATGTTTAAAGTTTTCC. These primers were designed to include a *PstI* site at the 5′-end and a *PvuI* at its 3′-end of the PCR fragment. The final DNA fragment was cloned in between the *PstI/PvuI* restriction sites of the modified pACYC184 plasmid. Insertions were confirmed by digesting with each restriction enzyme and by sequencing using the primer 5′-CACCGTCTTTCATTGCC-3’. Single changes were incorporated into the *tnaC* gene of the *tnaC tnaA’-‘camR* plasmid construct by using the Q5 site-directed mutagenesis kit and the primers shown in Table S2. The resulting plasmid constructs were selected with 10 μg/ml tetracycline (Tet).

### β-gal assays

β-gal assays (Tables 1 and S3–S5) were performed on cultures grown at 37 °C in M9 minimal medium (M9-MM) plus 0.2% glycerol, 0.05% casein acid hydrolyzed (ACH), 0.01% vitamin B1, and with or without the indicated amount of L-Trp as described before (25). Activity was defined in Miller units calculated as follows: 1000 X [*A*_420nm_ of the reaction to a given time/(*A*_600nm_ of culture sample X volume of culture sample (ml) X reaction time (min))].

### Chloramphenicol acetyltransferase assay

Assays were performed as follows: one bacterium colony was used to inoculate 1 ml M9-MM containing 0.5% ACH. Strains without plasmids were cultured without antibiotics while strains with pACYC184 and derivatives were selected by supplementing the growth media with 10 μg/ml Tet. Early the next day, 200 μl of overnight culture was used to inoculate 2 ml of M9-MM supplemented with 0.5% ACH, 10 μg/ml Tet, and with or without 25 μg/ml 1-methy-L-Trp (1MTrp). 1MTrp is an analog of L-Trp that is more stable *in vivo*. The new cultures were incubated at 37 ˚C with shaking (150 rpm) until the culture turbidity reached an *A*_600_ = 0.3. If cultures passed *A*_600_ ≥ 0.35 the densities were normalized by diluting them with fresh growth media. In all cases, the *A*_600_ was recorded and cells in 1 ml of the subculture were pelleted by centrifugation at 4 ˚C at 4000 rpm for 10 min (Eppendorf Centrifuge 5810R). The supernatant was discarded, and pellets were resuspended in 200 μl of a 100 mM Tris–HCl (pH 7.8) buffer solution. Next, the cells were lysed using a 4C15 sonicator (Thermo Fisher Scientific) at 50% amplitude for 10 s or until the solution was clear. Lysates were then stored on ice until needed. A Genesys20 Spec-10 spectrophotometer (Thermo Spectronic) was used to measure the change in absorbance (*A*_412_) of each reaction. To calibrate the spectrophotometer, a blank reaction was first assembled by combining in a cuvette 900 μl of 100 mM Tris–HCl (pH 7.8) buffer solution with 33 μl 2.5 mM 5,5’-dithio-bis(2-nitrobenzoic acid) (Sigma, Cat. no. D8130), 33 μl 5 mM acetyl-CoA (Sigma, Cat. no. A2056-10), and 17 μl 0.3% Cm. Test reactions were started by adding 5 μl lysates and absorbance was recorded every 10 s for at most 4 min. For each duplicate reaction, the average absorbance was used to normalize for differences in cell density by dividing the average *A*_412_ by the *A*_600_ of the subculture used. The normalized *A*_412_/*A*_600_ values were plotted against time to obtain Figure 2*A*, Figs. S2, S4, and S6.

### Cm inhibitory concentration assays

Inhibitory concentration assays were performed in 96-well plates using a range of Cm concentrations. Overnight cultures grown in M9-MM with 10 μg/ml Tet were diluted 4-fold with fresh media containing Tet. Later, 500 μl of these diluted cultures were mixed with 500 μl of fresh media containing Tet with or without 20 μM 1MTrp. Cells from the resulting mixtures were allowed to grow with aeration at 37 °C for 1 h to induce the expression of the *camR* reporter gene. Next, 150 μl of the resulting cultures were taken and placed in a well and mixed with 150 μl of fresh media containing Tet and with one of five different concentrations of Cm. Growth controls were mixed with fresh media containing Tet without Cm. The resulting mixes were incubated with aeration at 37 °C overnight growth and then the *A*_600_ for each well was measured for subsequent calculations. Recorded *A*_600_ were averaged for each strain and condition. The percent (%) average survival of samples at a given concentration of Cm observed in Figure 2, *B*–*D*, Figs. S1, S3 and S5 was determined using the following equation:

$$\%\;Avg.Survival=\left(\frac{Avg.\;survival\;at\;X\;\mu g/\;mL\;Cm}{Avg.\;survival\;at\;0\;\mu g/\;mL\;Cm}\right)\times 100$$

IC_50_ and IC_90_ values were estimated between the interval of Cm concentrations where 50% and 10% survival were observed, respectively (Table 2).

### Toeprinting assays

Toeprinting assays observed in Figure 3 were performed as described previously (2), In short, cell-free transcription-translation reactions using the PURExpress *in vitro* protein expression system (New England Biolabs, Cat. no. E6800S) were made with DNA templates obtained by PCR using *tnaC* (WT) plasmid pGF2500 or PGFS10P. The following primers were used: forward primer 5′-TAATACGACTCACTATAGGGAGTTTTATAAGGAGGAAAACATATGAATATCT TACATATATGTG-3′, to add the T7 promoter sequence and an optimized translation initiation region, and a reverse primer 5′-AGCAAACAAATAGATCACATTG-3′, which was also used as the toeprinting primer. Translation reactions, where the basal L-Trp concentration was 0.3 mM, were performed by adding extra L-Trp concentrations ranging from 0 to 25 mM. Products were then resolved on 6% urea/PAGE. Densities were determined using Fiji software (https://fiji.sc). Normalized density signals were obtained by dividing the density value of the L-Trp–dependent arrested signal by the density value of a signal band located 18-nt downstream from it. We selected the 18-nt downstream signal because it is not generated by the addition of L-Trp, and it is not affected as well by the arrest of the retro-transcriptase produced by ribosomes at the *tnaC* sequence. The “arrested ribosome fold accumulation” values used to make the plots shown in Figure 3*C* were calculated by dividing the normalized density of L-Trp–dependent arrested signals of each lane with the normalized density of the corresponding signal obtained in the absence of additional L-Trp (0 mM).

### Puromycin resistance assay

Puromycin resistance assays observed in Figure 4 were performed as follow: template PCR DNA was amplified from pGF2500, pGFS10P, or pGFR23F (∼600 base pairs), which contain either *tnaC* (WT), *tnaC*(*S10P*), or *tnaC*(*R23F*) sequences, as well as the *tna* UTR and a *rpoBC* terminator sequence (21). Template DNA for coupled transcription-translation *in vitro* reactions was amplified by PCR using the following primers: 5′-TAATACGACTCACTATAGGGAGTTTTATAAGGAGGAAAACATATGAATATCTTACATATATGTG-3′ and 5′-ACGGAATTCCTTGCCGAGTTTGACTC-3′. Biotinylated-mRNA transcripts were produced *in vitro* using T7 Promega RiboMAX Large Scale RNA Production Systems (Cat. no. P1300). Transcription reactions were performed as previously indicated (22) using 9 ng/μl PCR and 0.45 mM Biotin-16-UTP (Roche, Cat. no. 11388908910). The resulting biotinylated mRNA was verified *via* 1.5% agarose gel and analyzed using Fiji to determine the amount of mRNA present. One hundred microliters translation reactions were performed with cell-free extracts obtained as previously indicated (22), 0.5 μM biotinylated mRNA, and 0.5 μCi/μl [^35^S]-methionine ([^35^S]-Met) (10.25 mCi/ml; NEG709A, PerkinElmer) with 4 mM L-Trp. Arrested ribosomes attached to biotinylated mRNA were isolated using Promega Streptavidin MagneSphere Paramagnetic Particle beads (Cat. no. Z5481) as shown previously (22). Following final washing, beads were resuspended in 200 μl working solution containing 0.3 mM G418 sulfate (Geneticin by Gibco, Cat. no. 11811-023). G418 is a 30S binding aminoglycoside that blocks release factor binding without affecting puromycin action on resident peptidyl-tRNAs. Twenty microliters of isolated complexes were incubated with several concentrations of L-Trp at 37 ˚C for 5 min. This incubation step is needed to allow L-Trp to bind the ribosome before puromycin cleaves the TnaC-tRNA^Pro^ molecule. The reactions were split, and half were treated with puromycin and then incubated at 37 ˚C for ten more minutes. Reactions were stopped by adding an equal volume of 2X loading buffer and incubating at 70 ˚C for 5 min. Products were then resolved on 10% Tris-Tricine PAGE at 40 mA for 2.5 to 3 h. Separated products were then transferred to GE Whatman filter paper using a vacuum dryer. Dried gels were exposed and visualized on X-ray films. Densities were determined using Fiji. The values of % of [^35^S]-Met-TnaC as [^35^S]-Met-TnaC-tRNA^Pro^ were calculated as follow: [density of the [S^35^]-Met-TnaC-tRNA^Pro^ band/(density of the [S^35^]-Met-TnaC-tRNA^Pro^ + density of the [S^35^]-Met-TnaC)] X 100.

## Data availability

Additional data could be shared by contacting Luis R. Cruz-Vera at luis.cruz-vera@uah.edu, tel: (256) 824-6261.

## Supporting information

This article contains supporting information (21).

## Conflict of interest

The authors declare that they have no conflicts of interest with the contents of this article.
