# Supplementary material for: Functional domains of a ribosome arresting peptide are affected by surrounding nonconserved residues
Source: J Biol Chem. 2024 Feb 22;300(3):105780. doi: 10.1016/j.jbc.2024.105780 (PMC10941005; doi:10.1016/j.jbc.2024.105780)
Supplement: Supporting Information [file mmc1.docx]

**List of Data Included**

1. **Supporting Tables Page**

**Table S1** S-2

**Table S2** S-3

**Table S3** S-5

**Table S4** S-6

**Table S5** S-7

1. **Supporting Figures**

**Figure S1** S-8

**Figure S2** S-10

**Figure S3** S-11

**Figure S4** S-12

**Figure S5** S-13

**Figure S6** S-14

**Supporting Tables**

**Table S1.** Plasmids and bacterial strains used in this work.

| **Plasmid** | **Description** | **Source** |
| --- | --- | --- |
| pGF2500 | Contains the -160 to the +306 *tna* regulatory leader region fused to the rpoBC terminator incorporated into the pUC18 plasmid | 21 |
| pGFS10P | Derived from pGF2500 with the *tnaC* S10P change | This Study |
| pGFR23F | Derived from pGF2500 with the *tnaC* R23F change | This Study |
| pACY184 | GeneBank: X06403J |  |
| pHNG0 | Derived from pACY184; Contains PstI and PvuI sites upstream *camR* | This Study |
| pHNG1 | Derived from pHNG0; *tna_p_* *tnaC*; *tnaA'*-*camR* | This Study |
| pHNG2 | Derived from pHNG1; _10_TCA→CCA (S10P) | This Study |
| pHNG3 | Derived from pHNG1; _23_CGC→CAC (R23H) | This Study |
| pHNG4 | Derived from pHNG1; _12_TGG→CGG (W12R) | This Study |
| pHNG5 | Derived from pHNG4; _10_TCA→CCA (S10P/W12R) | This Study |
| pHNG6 | Derived from pHNG4; _23_CGC→CAC (W12R/R23H) | This Study |
| pHNG7 | Derived from pHNG1; _12_TGG→TTG (W12L) | This Study |
| pHNG8 | Derived from pHNG7; _10_TCA→CCA (S10P/W12L) | This Study |
| pHNG9 | Derived from pHNG7; _23_CGC→CAC (W12L/R23H) | This Study |
| pHNG10 | Derived from pHNG1; _12_TGG→GCC (W12A) | This Study |
| pHNG11 | Derived from pHNG10; _10_TCA→CCA (S10P/W12A) | This Study |
| pHNG12 | Derived from pHNG10; _23_CGC→CAC (W12A/R23H) | This Study |
| pHNG13 | Derived from pHNG1; _16_GAC→GAA (D16E)_ | This Study |
| pHNG14 | Derived from pHNG13; _10_TCA→CCA (S10P/ D16E) | This Study |
| pHNG15 | Derived from pHNG13; _23_CGC→CAC (D16E/R23H) | This Study |
| pHNG16 | Derived from pHNG1; _16_GAC→AAA (D16K) | This Study |
| pHNG17 | Derived from pHNG16; _10_TCA→ CCA (S10P/ D16K) | This Study |
| pHNG18 | Derived from pHNG16; _23_CGC→CAC (D16K/R23H) | This Study |
| pHNG19 | Derived from pHNG1; _16_GAC→GCC (D16A) | This Study |
| pHNG20 | Derived from pHNG19; _10_TCA→CCA (S10P/ D16A) | This Study |
| pHNG21 | Derived from pHNG19; _23_CGC→CAC (D16A/R23H) | This Study |
| pHNG22 | Derived from pHNG1; _24_CCT→TGC (P24C) | This Study |
| pHNG23 | Derived from pHNG24; _10_TCA→CCA (S10P/ P24C) | This Study |
| pHNG24 | Derived from pHNG24; _23_CGC→CAC (R23H/P24C) | This Study |

**Table S2.** Primers used in this work.

| **Mutation** | **Oligo. Name** | **Sequence (5’→3’)^1^** |
| --- | --- | --- |
| W12L | TnaCW12L_Q5_F | GACCTCAAAA**TTG**TTCAATATTGAC |
|  | TnaCW12L_Q5_R | ACACATATATGTAAGATATTCATAATG |
| S10P | WT_S10P_F | ATGTGTGACCCCAAAA**TGG**TTC |
|  | S10P_R | ATATGTAAGATATTCATAATGCAC |
| S10P/ W12L | W12L_S10P_F | ATGTGTGACCCCAAAA**TTG**TTC |
|  | S10P_R | *See above* |
| W12R | W12R_F | GACCTCAAAA**C**GGTTCAATATTG |
|  | TnaCW12L_Q5_R | *See above* |
| S10P/ W12R | W12R_S10P_F | A**C**GGTTCAATATTGACAACAAAATTGTC |
|  | W12R_S10P_R | TTTG**G**GGTCACACATATATGTAAG |
| S10P/ W12F | S10P/W12F_F | ACCCCAAAAT**TC**TTCAATATTGACAACAAAATTG |
|  | S10P/ W12F_R | CACACATATATGTAAGATATTCATAATG |
| W12A | W12A_fwd | GACCTCAAAA**GCC**TTCAATATTGACAACAAAATTGTC |
|  | TnaCW12L_Q5_R | *See above* |
| S10P/ W12A | S10P/ W12A_F | GACC**CCA**AAA**GCC**TTCAATATTGACAACAAAATTG |
|  | TnaCW12L_Q5_R | *See above* |
| D16E | D16E_F | GTTCAATATT**GAA**AACAAAATTGTCG |
|  | D16_Q5_R | CATTTTGAGGTCACACATATATG |
| S10P/ D16E | D16E_F | *See above* |
|  | S10P/D16_R | CATTTG**G**GGTCACACATATATG |
| D16E/ R23H | D16E_F | *See above* |
|  | D16_Q5_R | *See above* |
| D16A | D16A_F | GTTCAATATT**GCC**AACAAAATTGTCG |
|  | D16_Q5_R | *See above* |
| S10P/ D16A | D16A_F | *See above* |
|  | S10P/D16_R | *See above* |
| D16A/ R32H | D16A_F | *See above* |
|  | D16_Q5_R | *See above* |
| D16K | D16K_F | GTTCAATATT**AAA**AACAAAATTGTCGATCAC |
|  | D16_Q5_R | *See above* |
| S10P/ D16K | D16K_F | *See above* |
|  | S10P/D16_R | *See above* |
| D16K/ R23H | D16K_F | *See above* |
|  | D16_Q5_R | *See above* |
| R23H | R23H_F | ATTTTGTTGTCAATATTGAACCATTTTG |
|  | R23H_R | TGTCGATCAC**CAC**CCTTGATTTG |
| P24C | P24C_F | CGATCACCGC**TGC**TGATTTGCCC |
|  | P24C_R | ACAATTTTGTTGTCAATATTGAAC |
| R23H/P24C | R23FP24C_F | TGTCGATCAC**TTCTGC**TGATTTGCCCTTCTGTAG |
|  | R23FP24C_R | ATTTTGTTGTCAATATTGAACC |

^1^ Mutated sequence(s) are underlined and bolded.

**Table S3.** Translation of *tnaC(D16/R23H)* and *tnaC(R23H)* genes is required for suppression of LOF phenotype.

| **Strain** | **β-gal activity (MU)**^1^ | | | | **Induction ratio (+Trp/-Trp)**^2^ |
| --- | --- | --- | --- | --- | --- |
|  | **-Trp** | | **+Trp** | |  |
| **Wild-type** | 78 | ±17 | 3391 | ±179 | 43.5 |
| **D16E** | 31 | ± 4 | 51 | ± 6 | 1.6 |
| **R23H** | 521 | ±30 | 1690 | ±326 | 3.2 |
| **D16E/ R23H** | 99 | ± 6 | 512 | ± 43 | 5.2 |
| **Wild-type (∆AUG)** | 5 | ± 0 | 5 | ± 0 | 1.0 |
| **D16E (∆AUG)** | 5 | ± 0 | 5 | ± 0 | 1.0 |
| **R23H (∆AUG)** | 5 | ± 0 | 5 | ± 0 | 1.0 |
| **D16E/ R23H (∆AUG)** | 6 | ± 1 | 5 | ± 0 | 0.8 |

^1^Cultures of *rrn+ E. coli* bacterial strains AW153 (wild-type), AW643 (wild-type(ΔAUG)), AW821 (D16E), AW946 (D16E (ΔAUG)), AW922 (R23H), AW961 (R23H (ΔAUG)), AW925 (D16E/R23H), and AW973 (D16E/R23H (ΔAUG)) were grown in M9-MM plus 0.2% glycerol, 0.05% ACH 0.01% vitamin B1, with (+Trp) or without (-Trp) 100 μg/ml L-Trp. β-gal assays were performed in three independent experiments. ^2^ Induction ratio of values for cultures grown with L-Trp (+Trp) and those grown without L-Trp (-Trp).

**Table S4.** The ability of S10P to suppress the LOF D16E mutant is allele-specific.

| **Strain** | **β-gal activity (MU)**^1^ | | | | **Induction ratio (+Trp/-Trp)**^2^ |
| --- | --- | --- | --- | --- | --- |
|  | **-Trp** | | **+Trp** | |  |
| **Wild-type** | 52 | ± 2 | 2045 | ± 8 | 39.3 |
| **S10P** | 1016 | ±37 | 3196 | ±140 | 3.1 |
| **D16E** | 27 | ± 0 | 46 | ± 2 | 1.7 |
| **S10P/D16E** | 178 | ± 6 | 731 | ± 61 | 4.1 |
| **D16K** | 28 | ± 1 | 21 | ± 1 | 0.8 |
| **S10P/D16K** | 148 | ± 5 | 114 | ± 9 | 0.8 |
| **D16A** | 61 | ± 1 | 47 | ± 1 | 0.8 |
| **S10P/D16A** | 328 | ±41 | 373 | ± 37 | 1.1 |

^1^Cultures of *rrn*+ *E. coli* bacterial strains AW153 (wild-type), AW888 (S10P), AW821 (D16E), AW513 (D16A), AW797 (D16K), AW154 (W12R), AW909 (S10P/D16E), AW952 (S10P/D16A), and AW930 (S10P/D16K), and were grown in M9-MM plus 0.2% glycerol, 0.05% ACH, 0.01% vitamin B1, with (+Trp) or without (-Trp) 100 µg/ml L-Trp. β-gal assays were performed in three independent experiments. ^2^ Induction ratio of values for cultures grown with L-Trp (+Trp) and those grown without L-Trp (-Trp).

**Table S5.** The ability of R23H to suppress the LOF D16E mutant is allele-specific.

| **Strain** | **β-galactosidase activity (MU)**^1^ | | | | **Induction ratio (+Trp/-Trp)**^2^ |
| --- | --- | --- | --- | --- | --- |
|  | **-Trp** | | **+Trp** | |  |
| **Wild-type** | 68 | ± 3 | 2301 | ±82 | 33.8 |
| **R23H** | 1193 | ±43 | 2383 | ±83 | 2.0 |
| **D16E** | 28 | ± 2 | 51 | ± 2 | 1.8 |
| **D16E/R23H** | 187 | ± 8 | 510 | ±15 | 2.7 |
| **D16K** | 27 | ± 0 | 20 | ± 0 | 0.7 |
| **D16K/R23H** | 289 | ± 8 | 132 | ± 3 | 0.5 |
| **D16A** | 45 | ± 2 | 31 | ± 2 | 0.7 |
| **D16A/R23H** | 464 | ±52 | 403 | ±21 | 0.9 |

^1^Cultures of *rrn*+ *E. coli* bacterial strains AW153 (Wt), AW922 (R23H), AW821 (D16E), AW513 (D16A), AW797 (D16K), AW154 (W12R), AW925 (D16E/R23H), AW965 (D16A/R23H), and AW955 (D16K/R23H), were grown in M9-MM plus 0.2% glycerol, 0.05% ACH, 0.01% vitamin B1, with (+Trp) or without (-Trp) 100 µg/ml L-Trp. β-gal assays were performed in three independent experiments. ^2^ Induction ratio of values for cultures grown with L-Trp (+Trp) and those grown without L-Trp (-Trp).

**Supporting Figures**

**
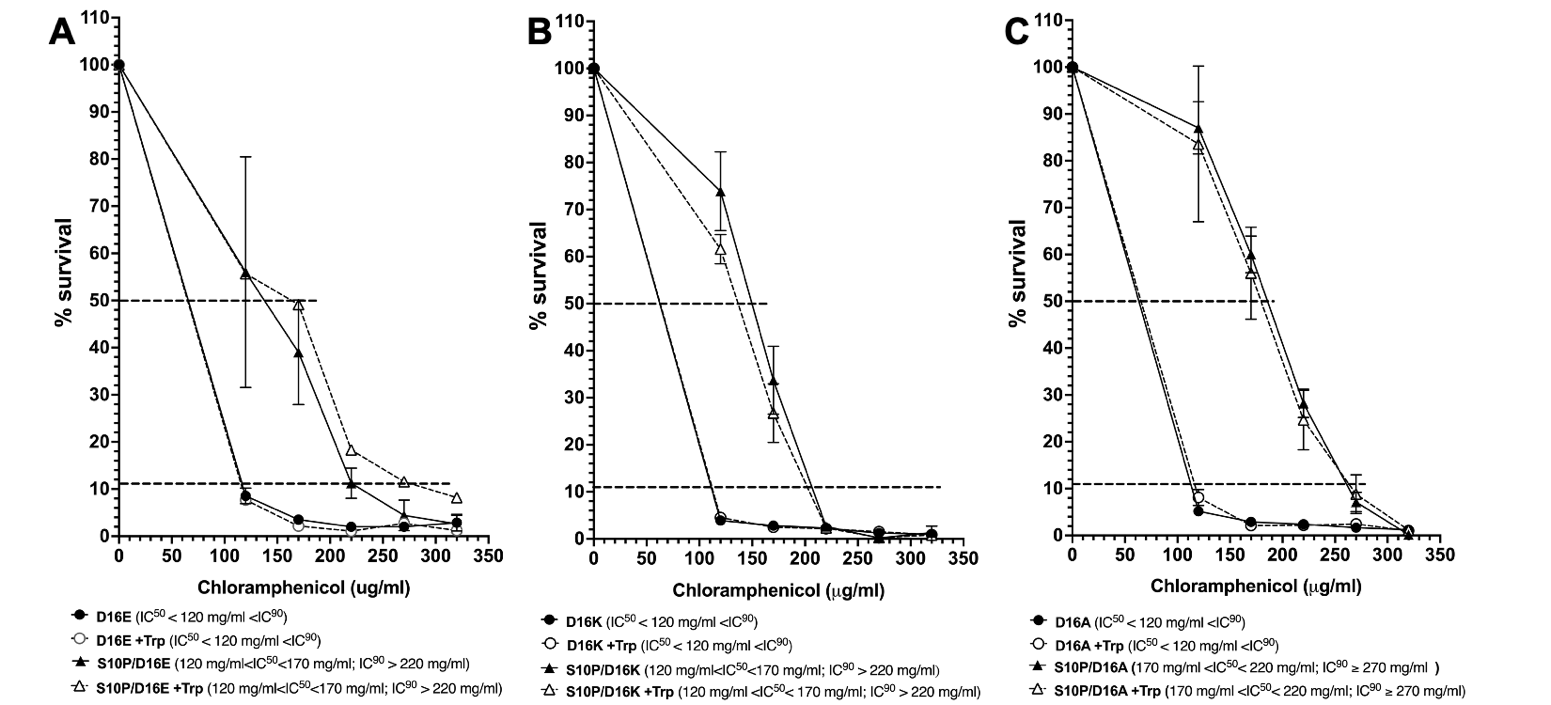
**

**
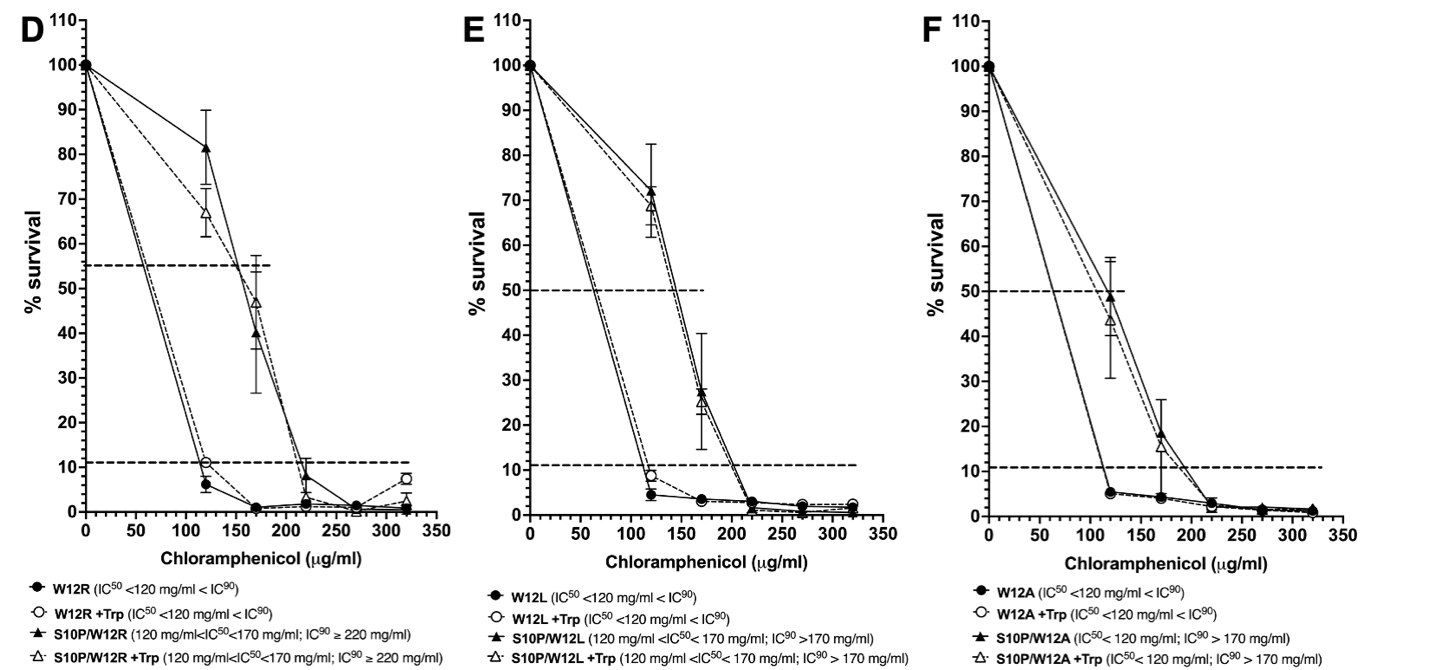
**

**Figure S1.** Survival of bacteria with *tnaC(S10P/D16X or S10P/W12X) tnaA’-‘camR* reporter gene fusion variants. Bacterial strains containing reporter genes with single (circles) and double (triangles) mutant changes at the *tnaC* gene were grown in MM9 without (open symbols) or with (closed symbols) 1MTrp and challenged with several concentrations of chloramphenicol. Dotted lines indicate the chloramphenicol concentration ranges were 50% and 10% survival were reached. Average survival ratios and standard deviation of the ratio of means of six independent experiments were calculated as indicated in Fig 1B. A) D16E and S10P/D16E, B) D16K and S10P/D16K, C) D16A and S10P/D16A, D) W12R and W12R/S10P, E) W12L and W12L/S10P, and F) W12A and W12A/S10P.

**
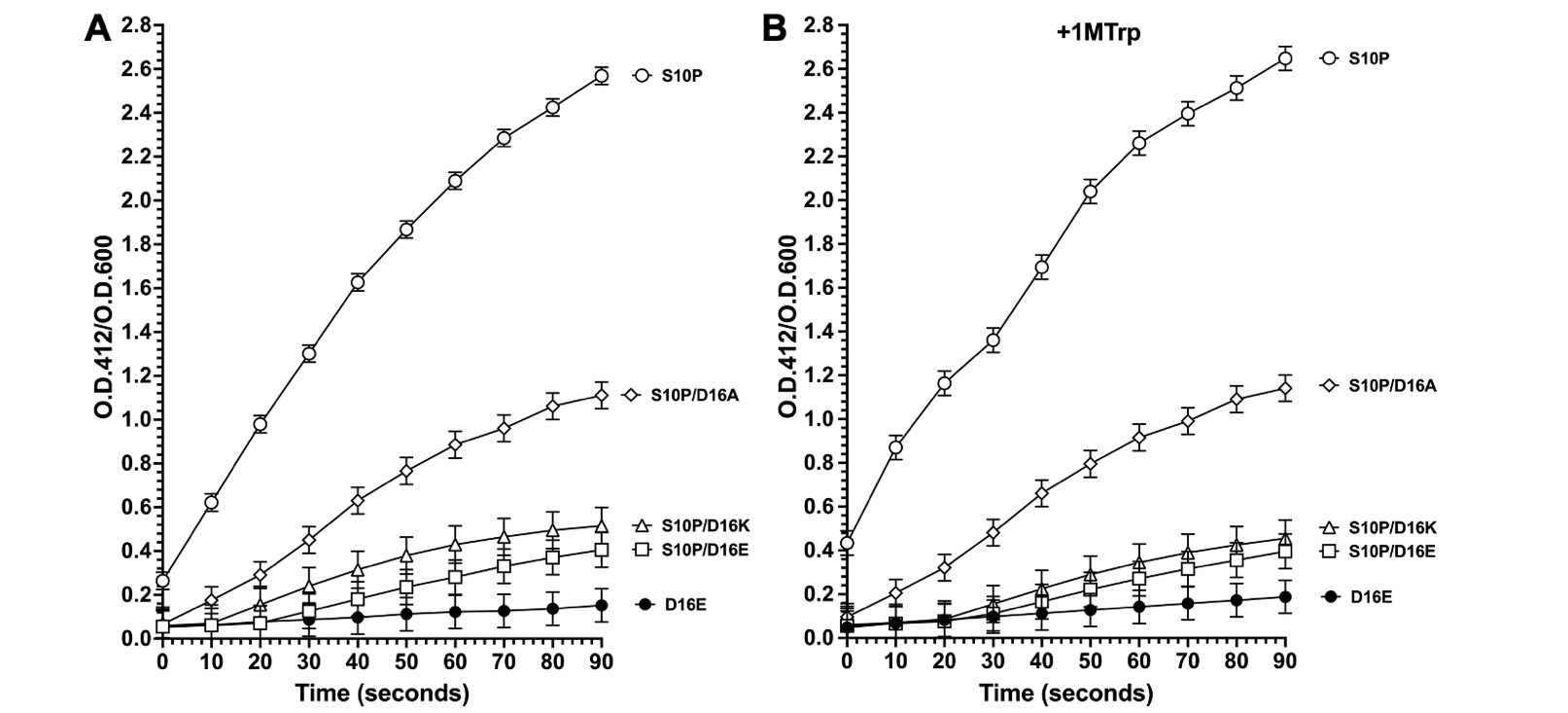
**

**
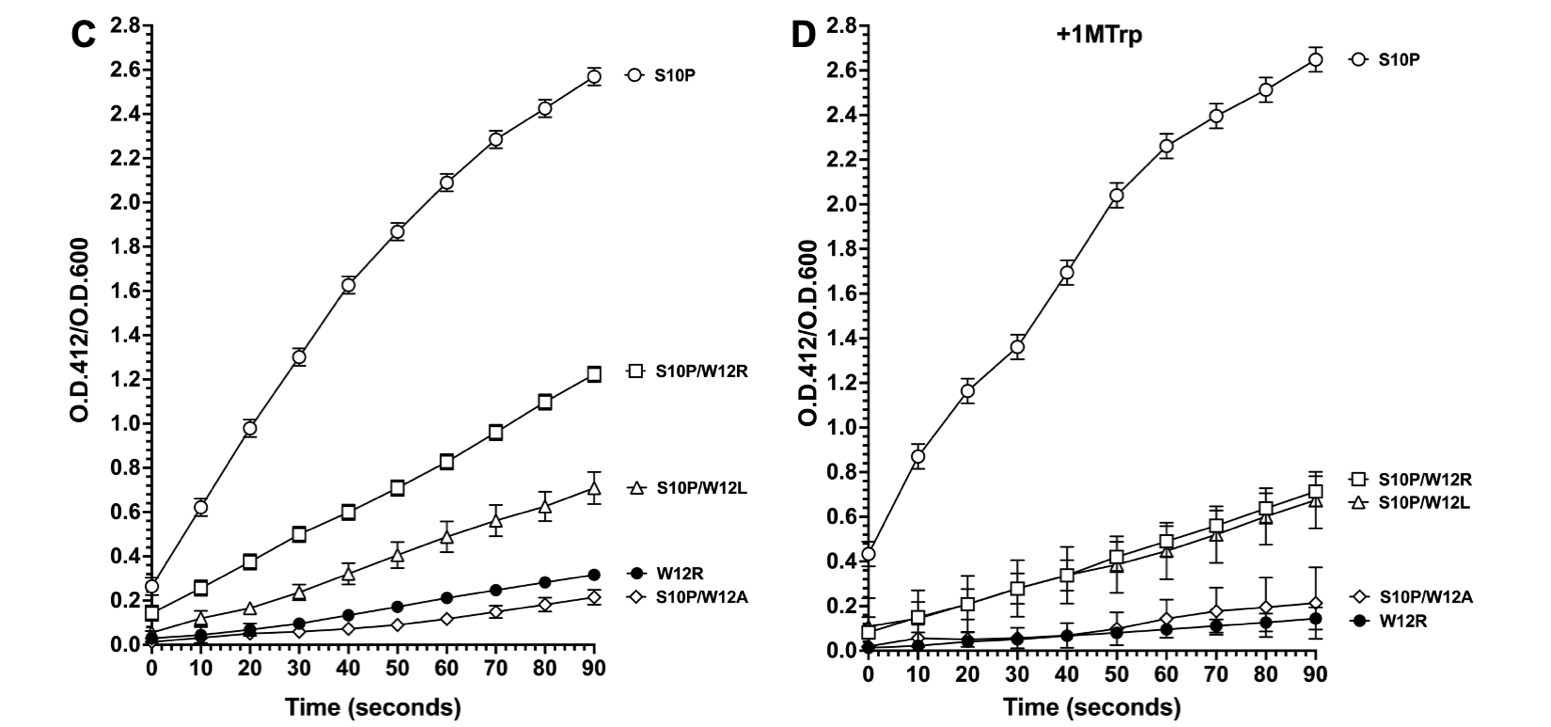
**

**Figure S2.** CAT enzymatic activity obtained from cells carrying *tnaC (S10P)-tnaA’-‘camR* mutant variants. Assays were performed in vivo as indicated in Figure 1A. Bars indicate the SEM of three independent experiments.

**
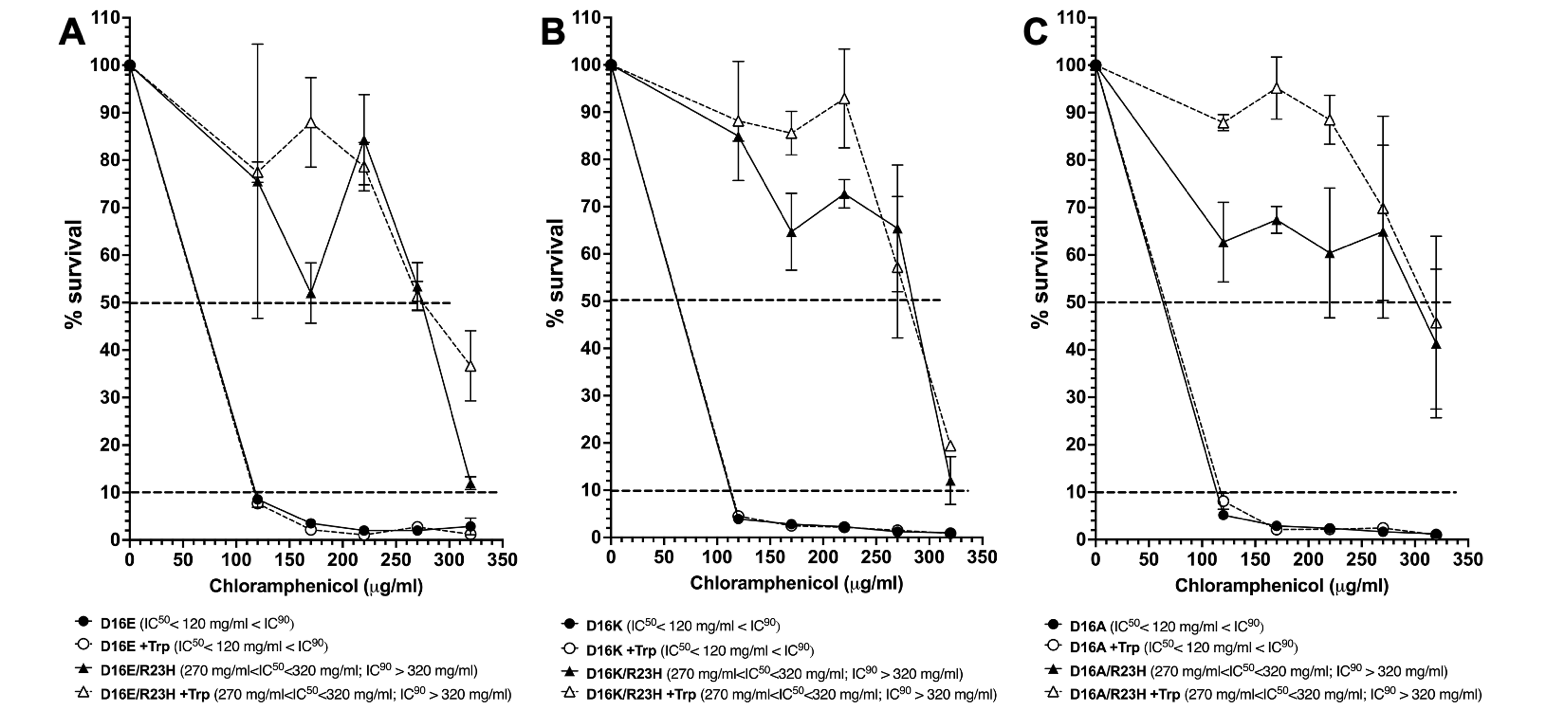
**

**
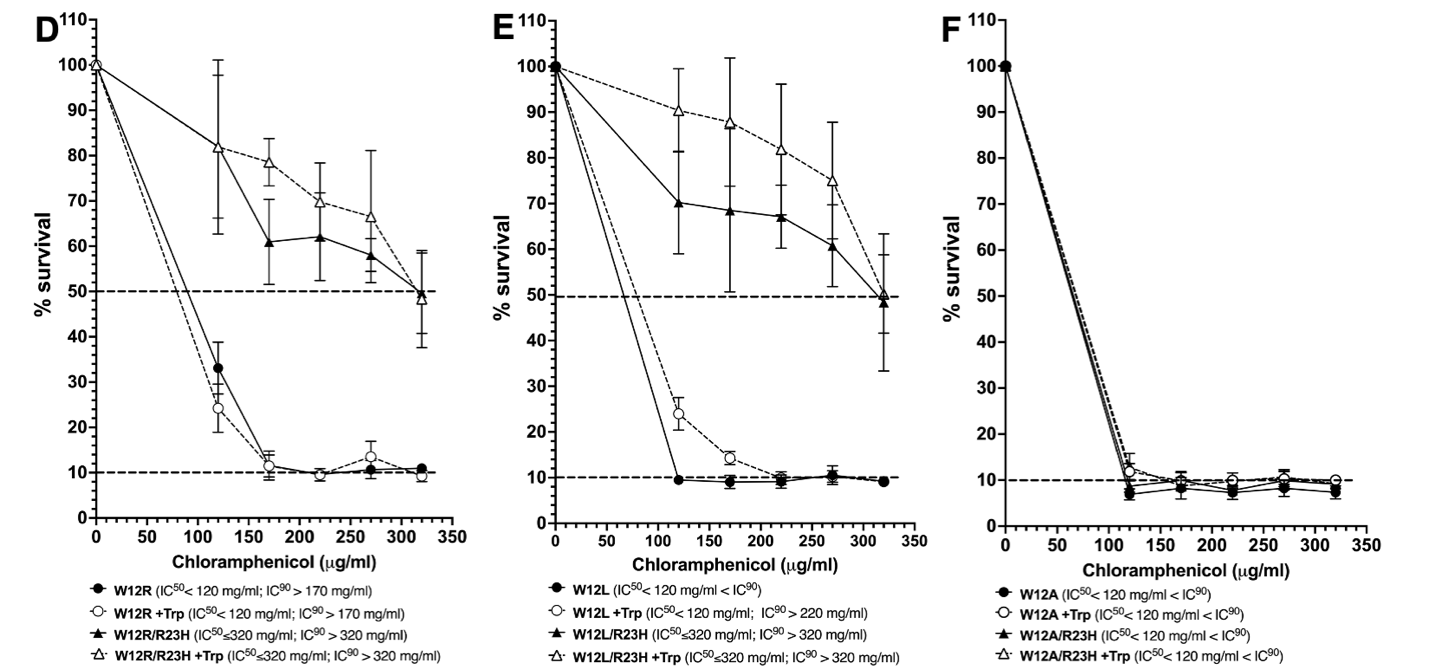
**

**Figure S3.** Survival of bacteria with *tnaC(D16X/R23H or W12X/R23H) tnaA’-‘camR* reporter gene fusions. Bacterial strains containing reporter genes with single (circles) and double (triangles) mutant changes at the *tnaC* gene were grown as indicated in Fig S1. A) D16E and D16E/R23H, B) D16K and D16K/R23H, C) D16A and D16A/R23H, D) W12R and W12R/R23H, E) W12L and W12L/R23H, and F) W12A and W12A/R23H.

**
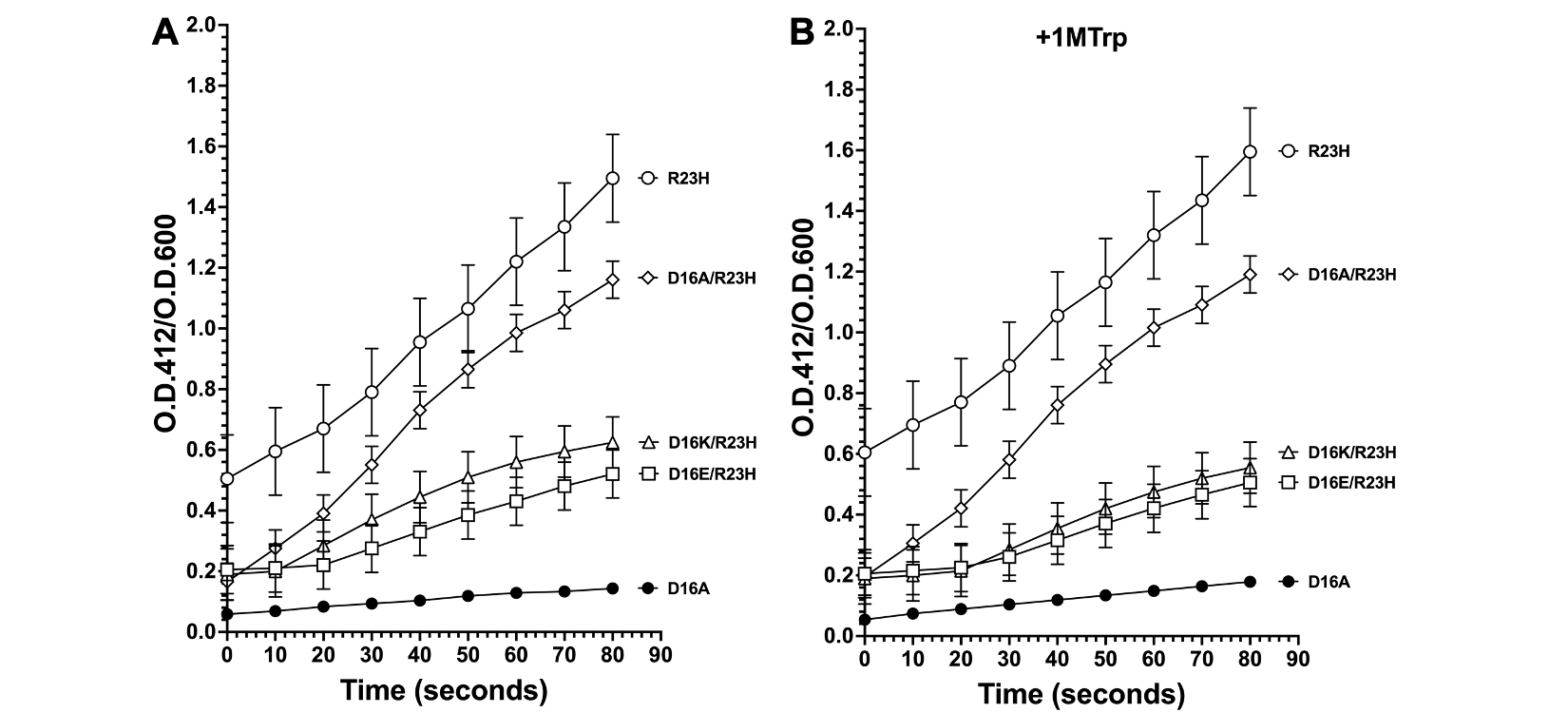
**

**
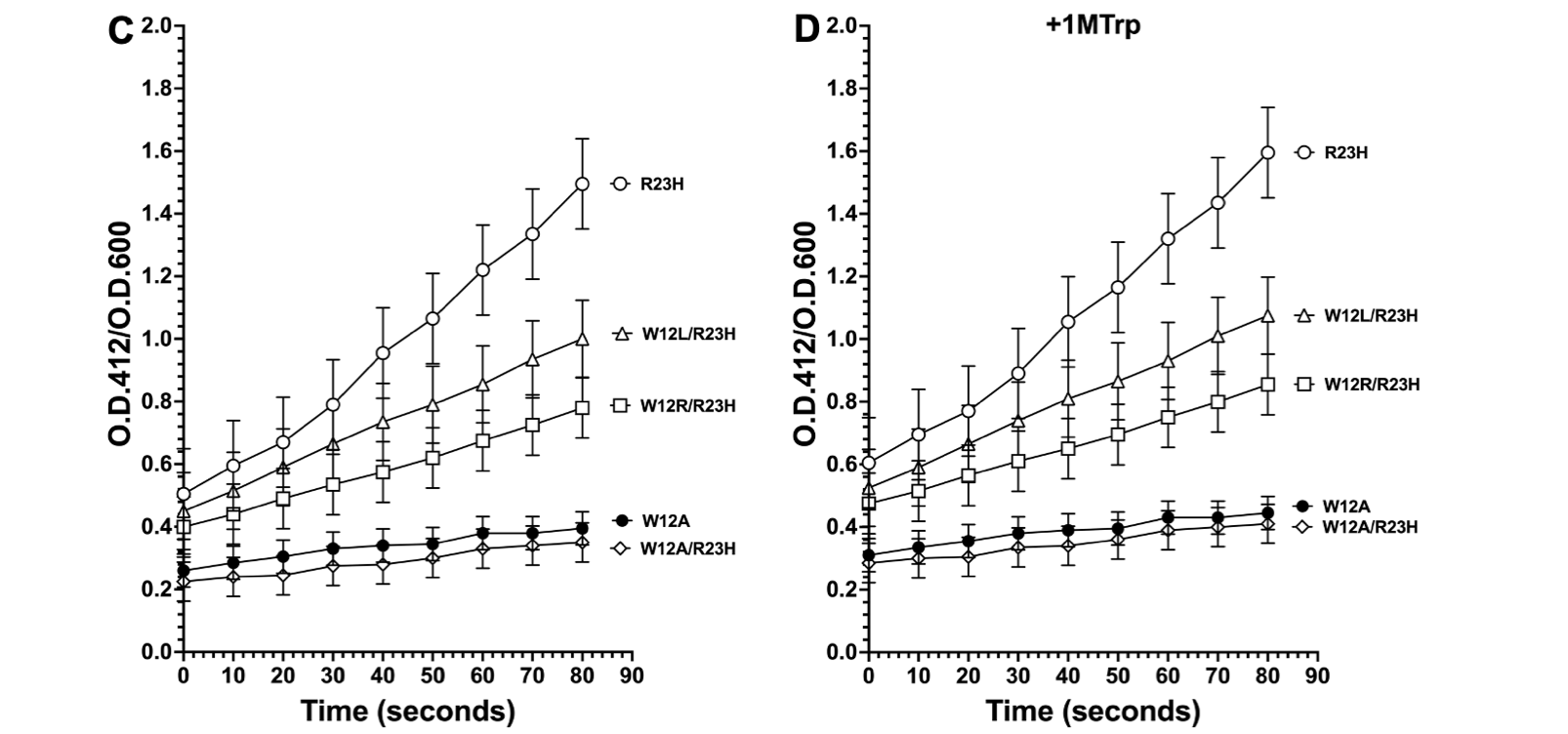
**

**Figure S4.** CAT enzymatic activity obtained from cells carrying *tnaC (R23H)-tnaA’-‘camR* mutant variants. Assays were performed in vivo as indicated in Fig S2. Bars indicate the SEM of three independent experiments.

**
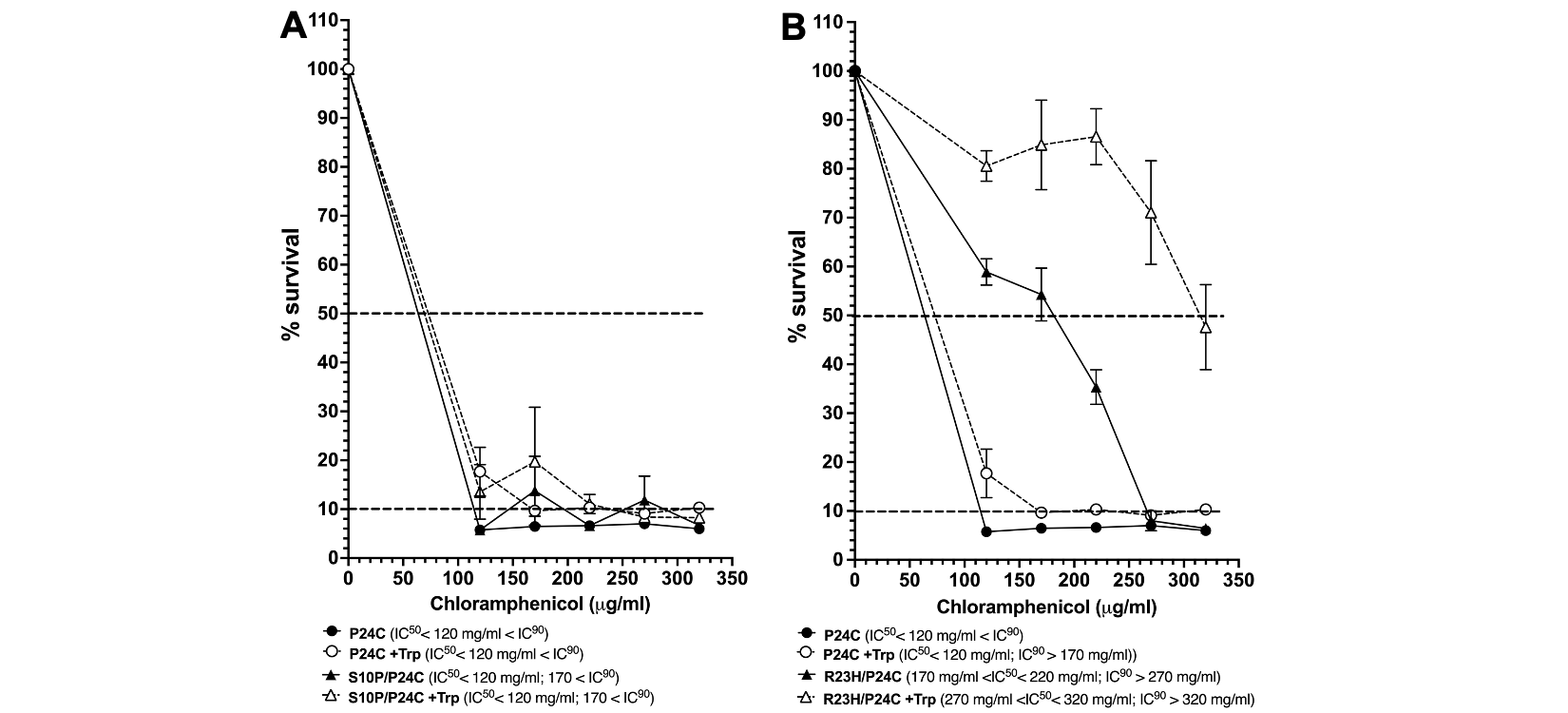
**

**Figure S5.** Survival of bacteria with *tnaC(S10P/P24C or R23H/P24C) tnaA’-‘camR* reporter gene fusions. Bacterial strains containing reporter genes with single (circles) and double (triangles) mutant changes at the *tnaC* gene were grown as indicated in Fig S1. A) P24C and S10P/P24C, B) P24C and R23H/P24C.

**
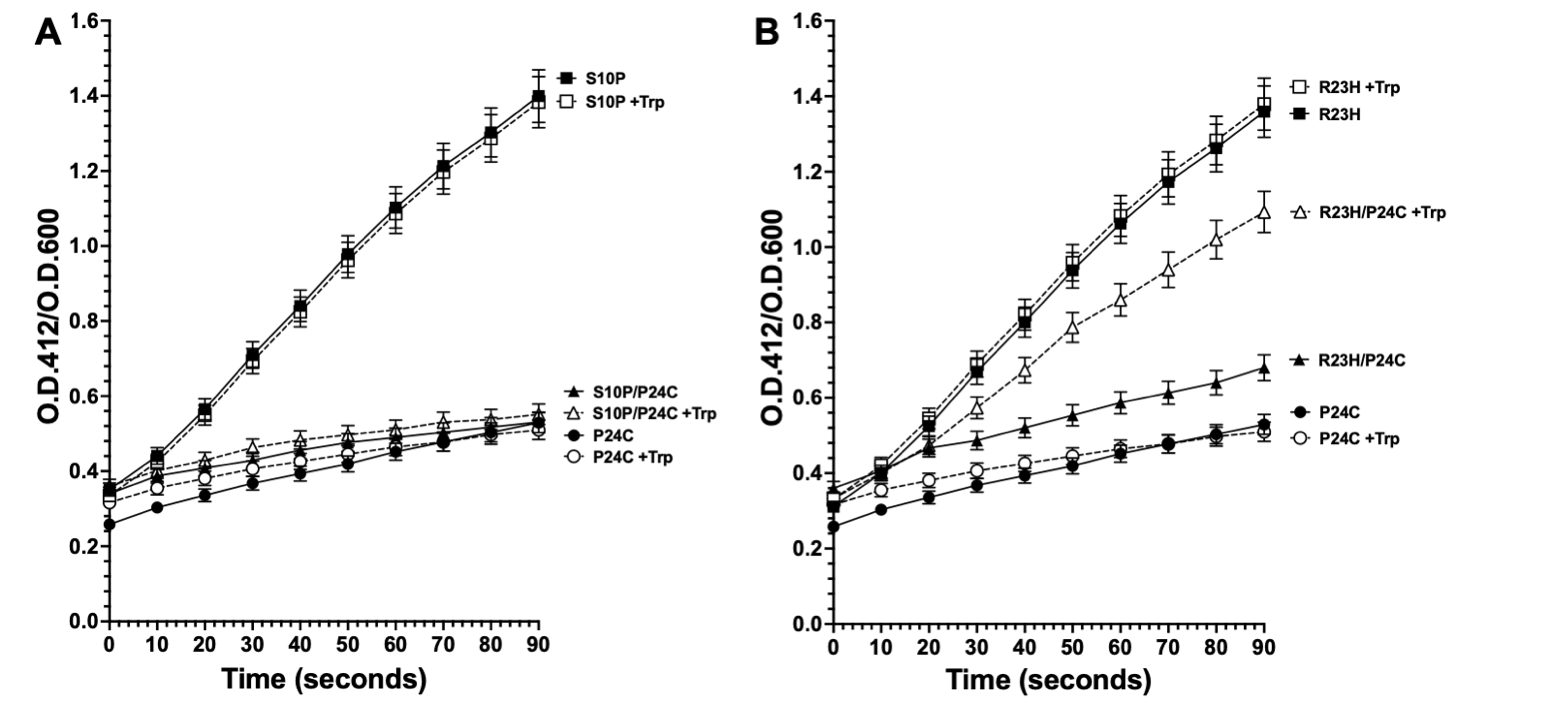
**

**Figure S6.** CAT enzymatic activity obtained from cells carrying *tnaC (P24C)-tnaA’-‘camR* mutant variants. Assays were performed in vivo as indicated in Fig S2. Bars indicate the SEM of three independent experiments.
